# Role of Bacterial and Host DNases on Host-Pathogen Interaction during *Streptococcus suis* Meningitis

**DOI:** 10.3390/ijms21155289

**Published:** 2020-07-25

**Authors:** Marita Meurer, Sophie Öhlmann, Marta C. Bonilla, Peter Valentin-Weigand, Andreas Beineke, Isabel Hennig-Pauka, Christian Schwerk, Horst Schroten, Christoph G. Baums, Maren von Köckritz-Blickwede, Nicole de Buhr

**Affiliations:** 1 Department of Physiological Chemistry, University of Veterinary Medicine Hannover, 30559 Hannover, Germany; Marita.Meurer@tiho-hannover.de (M.M.); Marta.Cristina.Bonilla.Gonzalez@tiho-hannover.de (M.C.B.); Maren.von.Koeckritz-Blickwede@tiho-hannover.de (M.v.K.-B.); 2 Research Center for Emerging Infections and Zoonoses (RIZ), University of Veterinary Medicine Hannover, 30559 Hannover, Germany; 3 Centre for Infectious Diseases, Institute of Bacteriology and Mycology, Faculty of Veterinary Medicine, University Leipzig, 04103 Leipzig, Germany; sophie.oehlmann@uni-leipzig.de (S.Ö.); Christoph.Baums@uni-leipzig.de (C.G.B.); 4 Department of Infectious Diseases, Institute for Microbiology, University of Veterinary Medicine Hannover, 30173 Hannover, Germany; peter.valentin@tiho-hannover.de; 5 Department of Pathology, University of Veterinary Medicine Hannover, 30559 Hannover, Germany; andreas.beineke@tiho-hannover.de; 6 Field Station for Epidemiology Bakum, University of Veterinary Medicine Hannover, 49456 Bakum, Germany; Isabel.Hennig-Pauka@tiho-hannover.de; 7 Department of Pediatrics, Pediatric Infectious Diseases, Medical Faculty Mannheim, Heidelberg University, 68167 Mannheim, Germany; Christian.Schwerk@medma.uni-heidelberg.de (C.S.); Horst.Schroten@umm.de (H.S.)

**Keywords:** *Streptococcus suis*, meningitis, DNase, neutrophils, NETs, pathogenesis

## Abstract

*Streptococcus suis* is a zoonotic agent causing meningitis in pigs and humans. Neutrophils, as the first line of defense against *S. suis* infections, release neutrophil extracellular traps (NETs) to entrap pathogens. In this study, we investigated the role of the secreted nuclease A of *S. suis* (SsnA) as a NET-evasion factor in vivo and in vitro. Piglets were intranasally infected with *S. suis* strain 10 or an isogenic *ssnA* mutant. DNase and NET-formation were analyzed in cerebrospinal fluid (CSF) and brain tissue. Animals infected with *S. suis* strain 10 or *S. suis* 10Δ*ssnA* showed the presence of NETs in CSF and developed similar clinical signs. Therefore, SsnA does not seem to be a crucial virulence factor that contributes to the development of meningitis in pigs. Importantly, DNase activity was detectable in the CSF of both infection groups, indicating that host nucleases, in contrast to bacterial nuclease SsnA, may play a major role during the onset of meningitis. The effect of DNase 1 on neutrophil functions was further analyzed in a 3D-cell culture model of the porcine blood–CSF barrier. We found that DNase 1 partially contributes to enhanced killing of *S. suis* by neutrophils, especially when plasma is present. In summary, host nucleases may partially contribute to efficient innate immune response in the CSF.

## 1. Introduction

Bacterial meningitis is a life-threatening disease [1] in humans and pigs that can be caused by *Streptococcus suis* [2,3]. The innate immune system immediately reacts against invading bacteria by recruiting neutrophils to the site of infection. Neutrophils are the major immune cell inside the cerebrospinal fluid (CSF) during bacterial meningitis [4]. In addition to phagocytosis, neutrophil granulocytes are known to release neutrophil extracellular traps (NETs), which consist of a DNA backbone decorated with several antimicrobial components [5] that serve to entrap and kill various pathogens. The host itself is known to produce DNases to recycle NET-structures and to avoid tissue-damaging effects of NETs during infectious or non-infectious diseases [6,7,8].

Various bacteria have evolved DNases as NET-evasion factors to escape from NET-entrapment and killing. For *S. suis* two NET-evading nucleases have been described: EndAsuis and SsnA. The membrane-anchored EndAsuis only contributes to NET-evasion. The released SsnA has additionally been shown to efficiently reduce antimicrobial effects of NETs to improve survival of *S. suis* in the presence of NETs [9,10]. Furthermore, SsnA is active in CSF during *S. suis* meningitis [2]. However, the question of whether SsnA contributes to the development of meningitis in piglets as the natural host in vivo has not been investigated so far.

Generally, the specific role of nucleases during bacterial infections is controversially discussed in the literature. A recent publication discussed the infusion of DNase 1 into the CSF [11] as a treatment opportunity during pneumococcal meningitis. The authors observed an enhanced bacterial clearance in the CSF of infected rats by increased phagocytosis and NET degradation when treated with DNase 1. In contrast to this protective effect of DNase 1 in meningitis, it was found that the respiratory tract pathogens *Actinobacillus pleuropneumoniae* and *Haemophilus influenzae* survive even better in the presence of neutrophils treated with DNases, because degraded NETs release nutrients as NAD, which enhance bacterial growth [12].

In this study, we investigated the role of the bacterial nuclease SsnA and the role of the host DNase 1 during *S. suis* meningitis in vivo and in vitro.

## 2. Results

### 2.1. SsnA Only Slightly Increases S. suis Virulence in Pigs

**Figure 1:**
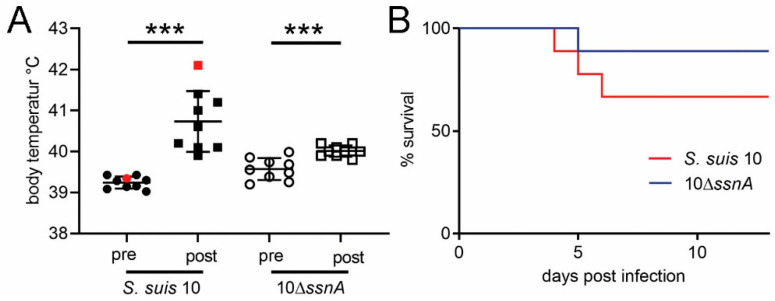
The DNase mutant *S. suis*10Δ*ssnA* suggest only a slight attenuation in pigs. The body temperature and survival rate after intranasal infection with *S. suis* is presented: (**A**) The body temperature increased in both infection groups post-infection. Data shown as mean ± SD. Each dot represents one animal, red marks show animals with meningitis. Statistical analysis: one-tailed, paired Student’s *t*-test was calculated in each infection group. Values post-infection present the highest measured value. *** *p* < 0.001. (**B**) The Kaplan–Meier survival curve shows a slightly higher but not significant mortality in the *S. suis* 10-infected group (Statistical analysis: Log-rank (Mantel–Cox) test, *p* = 0.26, each group *n* = 9).

The DNase SsnA is known as a major NET-evasion factor of *S. suis* [10]. To investigate the influence of the bacterial DNase SsnA during *S. suis* meningitis, we conducted two animal experiments. The first was performed as a survival experiment to examine the influence of the DNase on mortality over a period of 13 days. The second experiment was performed to study the immune response, as well as bacterial load, during the onset of meningitis (early phase). In the survival experiment, slightly higher internal body temperatures occurred in the *S. suis* 10-infected group than in the 10Δ*ssnA*-infected group (Figure 1A). In the *S. suis* 10-infected group, three out of nine animals died and in the 10Δ*ssnA* infected group one out of nine animals died (Figure 1B and Table S1). The pathohistological score and re-isolation of infection strains was almost similar in both infection groups (Tables S2 and S3) [13]. In the second experiment, we investigated the host–pathogen interaction during the onset of infection. Piglets were either infected with both infection strains or remained uninfected, and were euthanized after 48 or 96 h post-infection. Prior to the experiment, blood was taken and used for assaying the killing of *S. suis* 10 and 10Δ*ssnA*. Both infection strains showed similar survival rates in this assay (Figure S1). After intranasal infection, symptoms of CNS disorders were detected in four of 28 infected animals (*S. suis* 10: one pig; 10Δ*ssnA*: three pigs). A similar morbidity as in the initial survival experiment was found. The internal body temperature of infected animals increased significantly in both groups after infection and a slightly higher body temperature was detected in the *S. suis* 10-infected group post-infection (Figure S2 and Table S4). In piglets showing clinical signs of CNS disorders, the infection strains were re-isolated from the brain and in high numbers from the CSF (1.9 × 10^6^ to 3.7 × 10^7^ CFU/mL). The brain lesion score in this experiment was comparable between both infection groups (Tables S5 and S6). In summary, comparison of morbidity and mortality of both infection strains in both animal experiments leads to the conclusion that SsnA does not contribute to the development of meningitis in piglets. 

### 2.2. NETs Are Formed in CSF and Consist of PR-39 at the Onset of Meningitis

**Figure 2:**
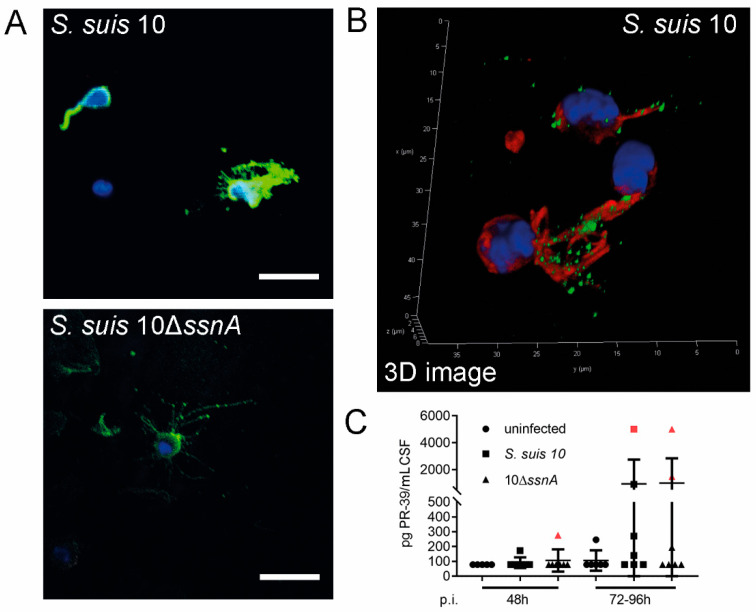
Neutrophil extracellular traps (NETs) are formed in the cerebrospinal fluid (CSF) of piglets infected with *S. suis* (strain 10) or its isogenic *ssnA* mutant and contain PR-39. Cytospins were conducted with CSF taken from infected piglets in the early phase experiment and were analyzed with immunofluorescence staining. (**A**) NET fibers were seen in CSF of piglets with meningitis from both infection groups (blue = DNA (Hoechst); green = DNA/histone-1-complexes (NETs)). Representative pictures are shown. (**B**) The antimicrobial peptide PR-39 is embedded in NET fibers and around the nucleus of neutrophils in CSF of infected piglets with meningitis from both infection groups. DNA/histone-1-complexes appeared to a lesser extent. The 3D image was modeled out of 50 z-stacks (0.17 µm steps) with LAS X 3D Version 3.1.0 software from Leica. (blue = DNA (Hoechst); green = DNA/histone-1-complexes (NETs); red = PR-39). Representative pictures are shown (scale bar A = 20 µm; B = 5 µm). (**C**) A high amount of antimicrobial peptide PR-39 in CSF of animals with clinical meningitis (red marks) was determined by ELISA. Data shown as mean ± SD. Statistical analysis: unpaired Mann–Whitney test.

In a previous study we analyzed pigs from a commercial herd with severe CNS disorders when suffering from *S. suis* meningitis and detected *S. suis* entrapped in NETs [2]. Furthermore, NETs were found in the CSF of experimentally *S. suis*-infected pigs with severe CNS disorders [2,14]. Here we were interested if NETs are also formed in the CSF at the onset of *S. suis* meningitis and analyzed CSF at defined time-points (48 and 96 h) post-infection. Indeed, we detected NETs in CSF in both infection groups (Figure 2A), indicating that SsnA does not completely destroy NETs in the CSF of infected piglets. Next we analyzed the amount of free DNA, described as a marker for NETs [15], to quantify the NET amount in the two infection groups (Figure S3). In general, and independent of the infection group, a higher amount of free DNA was detected in CSF compared to serum. Furthermore, NETs were released upon infection by infiltrating neutrophils in CSF in both infection groups in animals with CNS disorders, and no difference was detectable between *S. suis* 10- and 10Δ*ssnA*-infected piglets. Confirming our own previously published data on pigs from a commercial herd with severe CNS disorders [2], the antimicrobial peptide (AMP) PR-39 was detected in high amounts inside neutrophils in the CSF near the nuclei and in NET-fibers, together with DNA-histones (Figure 2B). AMPs are known to stabilize NETs against DNases and induce NETs in human and mouse neutrophils [16,17]. Interestingly, already in the onset of meningitis we could quantify a high amount of PR-39 and additional cationic antimicrobial peptides such as PMAP-23 or PMAP-37 in the CSF of piglets with clinical signs of CNS disorder (Figure 2C and Figure S4). These data indicate that during the onset of meningitis, NETs are formed as an innate immune response against *S. suis* invasion and are stabilized by host peptides such as PR-39 against degradation by *S. suis* nuclease.

### 2.3. NET-Markers Are Present in Meninges, but No NET-Fibers Are Detectable in Brain Tissue

**Figure 3:**
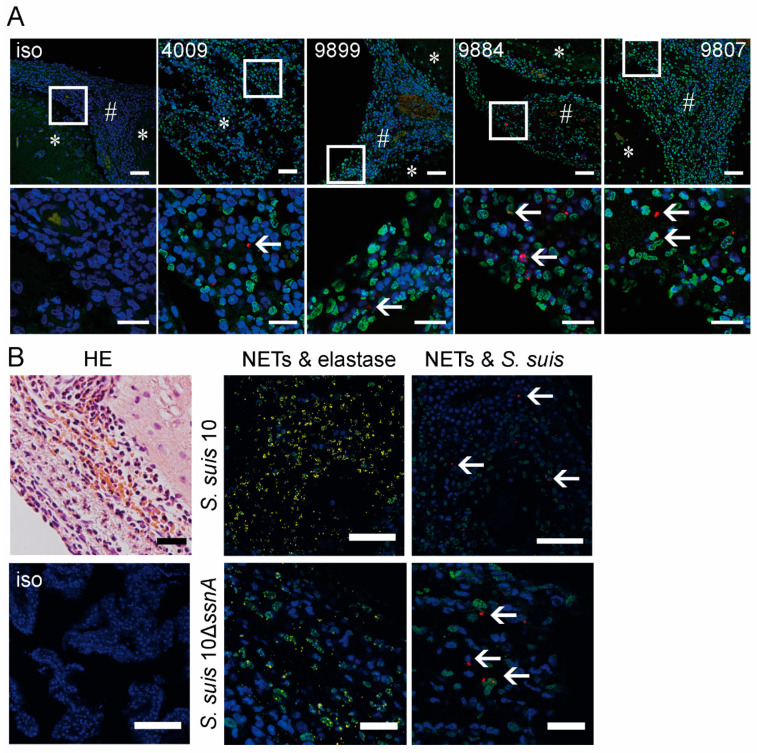
NET-markers are present in brain tissue of piglets with meningitis in situ, but no NET-fibers are detectable. (**A**) In the staining of formalin-fixed brain tissue from pigs out of survival experiments, NET markers and *S. suis* were visualized, but no NET fibers. * cortex cerebri and # inflammation in meninges (blue = counterstaining of DNA (DAPI); green = DNA/histone-1-complexes (NETs); red = *S. suis* (white arrow); iso = isotype control; 4009 = animal with meningitis from this survival experiment study; 9899, 9884 and 9807 = pigs with meningitis from other survival experiment study [2,14] (scale bar upper panel = 50 µm; lower panel = 20 µm). (**B**) Representative hematoxylin-eosin (HE) stained tissues of meningitis show neutrophils invading from vessels into the meninges. This picture was observed in both infection groups of the early phase experiment. NET markers (green = DNA/histone-1-complexes (NETs) and yellow = neutrophil elastase) and *Streptococci* (red) were found in brain tissue of these animals in both infection groups; however, as in the survival experiment, no NET fibers were detectable. Representative pictures are shown; white arrows = *S. suis*. The pictures for both infection groups present slides that were sliced successively (scale bar HE, isotype and *S. suis* 10 = 50 µm, *S. suis* 10Δ*ssnA* = 20 µm). Respective isotype controls were used to adjust the settings.

In the case that *S. suis* is not efficiently eliminated from the CSF by the host, it has the ability to infect the meninges [18]. Accordingly, *S. suis* was re-isolated from brain swap and CSF of a pig with meningitis (pig4009, Figure 3A). To protect the host against further pathogen dissemination into the brain, neutrophils infiltrate into the meninges. We wondered whether NETs could be detected in meninges and the choroid plexus, which is described as one of the entry sites of *S. suis* into the CNS [19,20,21]. By immunofluorescence staining of three different brain regions that contain meninges or choroid plexus (Figure S5), we visualized *S. suis* and NET markers (Figure 3).

**Figure 4:**
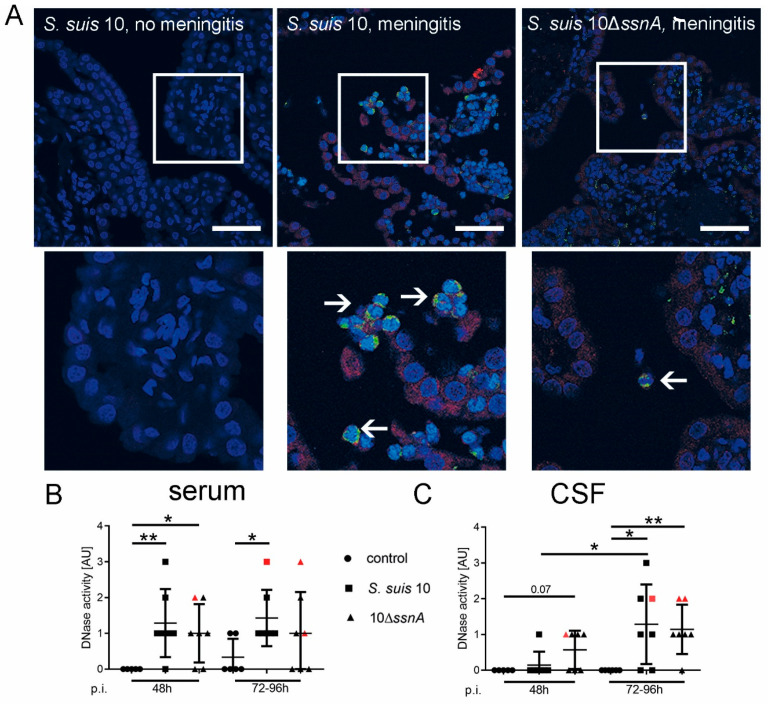
Host DNases are detectable and active during *S. suis* meningitis in choroid plexus, CSF and serum. (**A**) By immunofluorescence staining, we could show that the amount of DNase 1 in the choroid plexus is different in animals with inflammation, compared to animals without inflammation of the choroid plexus. In the choroid plexus of an infected pig without clinical signs of CNS disorders only weak DNase 1 signals were detected in the early phase experiment. In pigs with clinical signs of CNS disorders a high DNase 1 signal in the region of the choroid plexus epithelial cells was observed. PR-39 signal, as a marker for neutrophils, was found in these pigs as well (blue = DNA (Hoechst); green = PR-39; red = DNase 1 (scale bar upper panel = 50 µm; lower panel = 20 µm; arrows mark transmigrating or already transmigrated PR-39 positive neutrophils). Activity of DNases was observed in serum (**B**) and in CSF (**C**) of all pigs of the early phase experiment. A significant increase of DNase activity post-infection was found in serum for both infection groups after 48 h and for the *S. suis* 10-infected group also after 96 h. In CSF after 96 h, a higher increase was observed than after 48 h in both infection groups. Data shown as mean ± SD. Statistical analysis: unpaired Mann–Whitney test (* *p* < 0.05, ** *p* < 0.01).

In the case of the early phase experiment, we analyzed serial cuts, and DNA-histone and elastase as NET-markers, as well as *S. suis*, could be detected in both infection groups linked to neutrophils in the meninges. Importantly, no NET fibers were detectable. Hematoxylin-eosin (HE)-staining identified neutrophils in the choroid plexus and meninges only in animals with CNS disorders (Figure 3B and Figure S6). In good correlation to findings in the CSF, we found PR-39-positive neutrophils in the choroid plexus. Nevertheless, PR-39 was only detectable inside neutrophils and no NET fibers as shown in CSF were detectable (Figure 4A and Figure S7). In animals without symptoms of CNS disorders, we could neither detect PR-39 signals nor neutrophils in the brain. Fibers seen in HE-staining are not positive for DNA-histone, elastase or PR-39, and may therefore consist of fibrin instead of NETs.

Based on this finding that NET fibers occur in CSF but not in the meninges of both infection groups, we conclude that the bacterial DNase SsnA does not play a major role in the destruction of NETs in this immune-privileged compartment. Thus, we assume that the host itself seems to be responsible for modulating the formation and destruction of NETs by the production of host DNases.

### 2.4. Host DNases Are Active during S. suis Meningitis in Brain Tissue, CSF and Serum

Host DNases eliminate NETs to avoid tissue damage [8]. To prove our hypothesis of the involvement of host DNases during *S. suis* meningitis, we analyzed brain tissue by immunofluorescence microscopy. More DNase 1 signaling was detected in the choroid plexus of animals with clinical meningitis than in those animals without clinical meningitis (Figure 4A).

To generate quantitative data, we analyzed serum and CSF for DNase activity (Figure 4B,C). In serum was almost no DNase activity in the control group, but significantly higher DNase activity was detectable in both infection groups (Figure 4B), independent of whether *S. suis* 10 or 10Δ*ssnA* was used to infect the animals. Similar in CSF no nuclease activity was detected in the control group (Figure 4C) but significantly higher DNase activity was detected 96h post-infection in both the CNS. *S. suis* 10 infected animals sacrificed 96h post-infection had a significantly higher DNase activity than the group euthanized after 48h (Figure 4C and Figure S8). High amounts of specific DNase 1 were detected in animals with clinical signs of CNS disorders 96 h post-infection in CSF by ELISA (Figure S9).

The in vivo experiments indicate a minor influence of the bacterial DNase on the host–pathogen interaction during *S. suis* meningitis. To understand the role of host DNases, we conducted in vitro experiments.

### 2.5. DNase 1 Has No Impact on Transmigration of Neutrophils through a Choroid Plexus Epithelial Cell Layer

**Figure 5:**
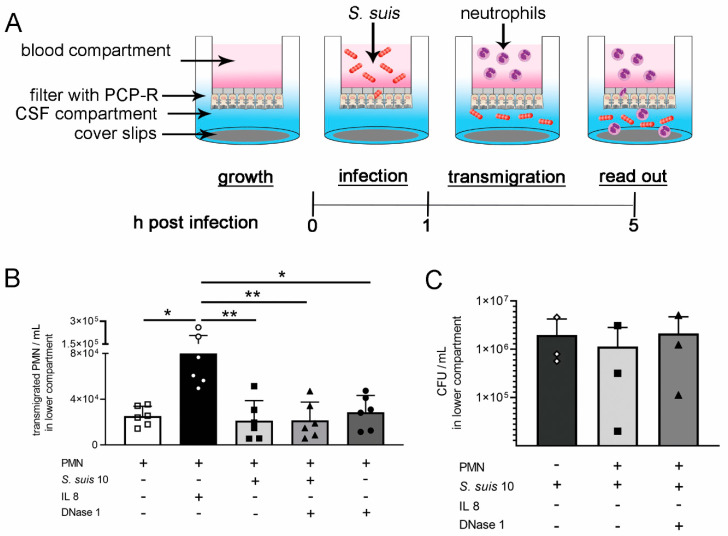
DNase 1 has no impact on transmigration of isolated porcine neutrophils through a cell layer of porcine choroid plexus epithelial cells and killing of *S. suis* by neutrophils. (**A**) An inverted cell culture model with porcine choroid plexus epithelial (PCP-R) cells was used to investigate transmigration of neutrophils after infection with *S. suis*. PCP-R cells were grown on the underside of a filter insert with 3 µm pores. *S. suis* 10 transmigrated from the blood compartment at the basolateral side into the medium-filled CSF compartment at the apical side of PCP-R cells. The medium with bacteria in the blood compartment was removed and replaced by medium containing freshly isolated porcine neutrophils (polymorph nuclear cells (PMN)). Neutrophils were allowed to transmigrate through the cell layer for 4 h and counteract against *S. suis* in the CSF compartment. As read-out neutrophil/mL and CFU/mL were determined. (**B**) Stimulation with 100 ng/mL interleukin 8 (IL8) leads to high transmigration of neutrophils into the CSF compartment in the cell culture system. (**C**) The CFU/mL was not significantly influenced by transmigrated neutrophils with or without DNase 1 treatment. (+) component present in the well, (−) component not present in the well. Data shown as mean ± SD; *n* = 3 independent experiments in duplicates. Statistical analysis: one-way ANOVA followed by Kruskal–Wallis multiple comparison test (* *p* < 0.05, ** *p* < 0.01).

The blood–CSF barrier is described for *S. suis* as an entry site [21,22]. We investigated the role of host DNase 1 on host–pathogen interaction using a model of the porcine blood–CSF barrier [23]. This model was adapted for *S. suis* 10 infection and neutrophil transmigration (Figure 5A). The barrier integrity was confirmed by transepithelial electrical resistance (TEER) measurement and dextran flux (Figure S10). Neutrophils spontaneously transmigrated through this barrier and interleukin 8 (IL8) enhanced the transmigration of neutrophils (Figure 5B). In contrast to IL8, *S. suis* 10 or DNase 1 or a combination of the two, did not increase the transmigration rate under the chosen 4-h incubation time (Figure 5B and Figure S11). There was no efficient elimination of bacteria detectable whether DNase 1 was present or not (Figure 5C).

**Figure 6:**
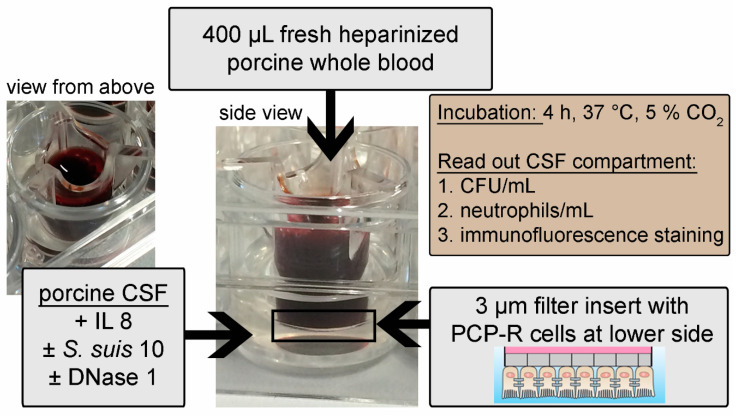
Cell culture model of the porcine blood–CSF barrier mimicking physiological conditions to investigate infection and transmigration. Neutrophils transmigrate out of fresh heparinized porcine whole blood through a layer of porcine choroid plexus epithelial cells (PCP-R) into porcine CSF. The CSF was enriched with interleukin 8 (IL8) to attract neutrophils. In addition, combinations with *S. suis* 10 infections and DNase 1 treatments were used. After 4 h of incubation, CFU/mL was determined by plating serial dilutions on blood agar plates and the number of transmigrated cells was determined by flow cytometry. Immunofluorescence staining of transmigrated neutrophils was conducted to analyze NET formation.

**Figure 7:**
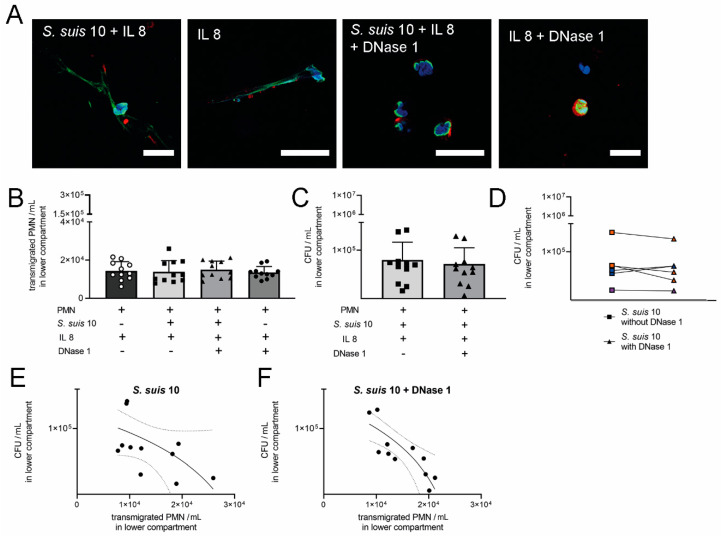
DNase 1 has no impact on transmigration of neutrophils trough a cell layer of PCP-R and killing of *S. suis* is donor-dependent. (**A**) NET formation was detectable in the CSF compartment in the porcine blood–CSF barrier mimicking physiological conditions in the absence of DNase 1, but was completely destroyed by DNase 1 (blue = DNA (Hoechst); green = DNA/histone-1-complexes (NETs); red = elastase). Representative pictures are shown (scale bar = 20 µm). (**B**) Transmigration of neutrophils out of fresh porcine blood trough PCP-R was neither significantly influenced by *S. suis* 10 nor DNase 1. Data shown as mean ± SD, *n* = 6 independent experiments with five duplicates and once a single value. Statistical analysis one-way ANOVA. (**C**) Killing of *S. suis* 10 in CSF was not significantly influenced by transmigrated neutrophils in presence of DNase 1. Statistical analysis: unpaired Student’s *t*-test. Data shown as mean ± SD, *n* = 6 independent experiments with five duplicates and once a single value. (**D**) Values from one experiment, with blood from the same donor pig, without and with DNase 1 in the CSF compartment are connected by a line. A donor-dependent influence regarding the effect of DNase 1 on killing efficiency is detectable (orange = DNase 1 reduces CFU; blue = DNase 1 increases CFU; violet = DNase 1 no impact on CFU). (**E**) Correlation of transmigrated neutrophils and *S. suis* 10 in CSF. The more neutrophils transmigrate into the CSF, the higher is the killing of the bacteria. Statistical analysis: Pearson correlation *r* = −0.5. (**F**) Correlation of transmigrated neutrophils and *S. suis* 10 + DNase 1 in CSF. With DNase 1 the Pearson correlation *r* = −0.8 is significant, *p* = 0.0026. E and F are calculated based on data from B and C.

As a further optimization of this model to mimic the in vivo situation as close as possible, we used whole blood in the upper blood-compartment and porcine CSF in the lower CSF-compartment instead of medium (Figure 6). The barrier integrity (Figure S12) and NET formation were confirmed using this optimized model (Figure 7A).

Again, using this optimized model, the transmigration rate of neutrophils into the IL8 stimulated CSF-compartment was not affected by DNase 1 (Figure 7B). However, the number of transmigrated neutrophils seems to depend on the individual blood donor, i.e., characteristic or number of neutrophils present in the blood.

### 2.6. DNase 1 Partially Improves Killing of S. suis by Neutrophils of Individual Animals in CSF

As DNase 1 did not increase neutrophil transmigration, we analyzed if DNase 1 might influence the antimicrobial activity of the neutrophils as previously shown [11]. Interestingly, in 50% of the blood donors an increased killing of *S. suis* 10 was detectable in the presence of DNase 1; however, this difference was not statistically significant (Figure 7C,D). A statistical correlation between transmigrated neutrophils and bacteria revealed the following phenomenon: the more neutrophils migrated through the barrier, the fewer bacteria survived in the CSF. Importantly, this correlation was only significant in the presence of DNase 1 (Figure 7E,F). Thus, we assume that DNase 1 might partially enhance the killing activity of neutrophils.

**Figure 8:**
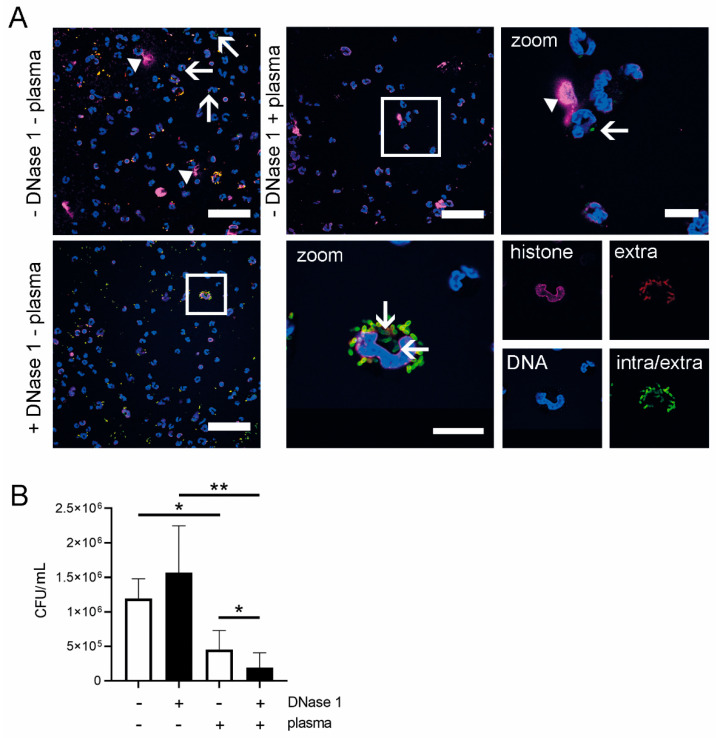
DNase 1 improves killing of *S. suis* by neutrophils in the presence of plasma. (**A**) Immunofluorescence staining of NETs and intra- and extracellular *S. suis*. NET production and phagocytosis exist in parallel during neutrophil killing assay. With plasma, less bacteria can be seen than without plasma. More *Streptococci* are extracellular than intracellular as the single channels visualize (blue = DNA (Hoechst); magenta = DNA/histone1-complexes (NETs, white arrowheads); red and green = extracellular *S. suis*; green = intracellular *S. suis* (white arrows)). Representative pictures are shown (scale bar = 50 µm, zoom = 10 µm). (**B**) The effect of DNase 1 on killing of *S. suis* 10*cps*ΔEF was proven with a neutrophil killing assay with or without 10% plasma of the blood-donating pig. After 2 h incubation with plasma, an increased killing of *S. suis* was detectable with DNase 1. Without plasma, the CFU/mL was significantly higher than with plasma. The best killing effect was detectable with plasma and DNase 1. Data shown as mean ± SD, *n* = 5 independent experiments. Statistical analysis: paired students *t*-test (* *p* < 0.05, ** *p* < 0.01).

To confirm the influence of DNase 1, we have chosen a more sensitive in vitro system and performed a neutrophil killing assay with the phagocytosis-sensitive *S. suis* capsule mutant (10*cps*ΔEF) [24,25]. Using immunofluorescence staining, neutrophils were confirmed to phagocytose and to release NETs in the same experiment (Figure 8A). Without plasma (absence of opsonization factors) a tendency for less growth inhibition in the presence of DNase 1 was detected, indicating NET-mediated killing in the absence of plasma. In the presence of 10% autologous plasma a significant decrease of bacteria was detected with DNase 1 compared to neutrophils without DNase 1 treatment (Figure 8B). These data confirm that DNase 1 contributes to enhancement of neutrophil antimicrobial activity, as previously shown [11], but only in the presence of autologous plasma.

## 3. Discussion

The aim of our study was to investigate the role of the bacterial nuclease SsnA and the host DNase 1 during *S. suis* meningitis in vivo and in vitro. Bacteria developed nucleases in evolutionary biology for phage defense [26]. Since the principle of defense by DNA destruction was very effective, it can be hypothesized that it was also adopted by mammalian cells or perhaps even transferred from bacteria into these cells by endosymbiosis; for example, mitochondrial endonuclease G, which has homologous nucleases in bacteria [27]. Homologous nucleases of SsnA also occur in other bacterial species such as *Streptococcus mutans*, *Streptococcus pyogenes*, *Bacillus anthracis*, *Vibrio cholerae* or *Aeromonas hydophila* [28]. In in vitro experiments, SsnA is described as a NET evasion factor and the strongest phenotype was found with human neutrophils [10]. Most *S. suis* field isolates found in inner organs of pigs express SsnA [28]; therefore, we analyzed if SsnA is necessary to cause meningitis in piglets in vivo. Two independent infection studies with piglets showed that the SsnA mutant was as virulent as the wild-type strain (Figure 1 and Figure S2). Several animals in both infection groups from the early phase experiment developed clinical signs of CNS disorders between 48 h and 96 h post-infection. In addition, *S. suis* strains were re-isolated from different inner organs of piglets without CNS disorders (Tables S3 and S6). In these piglets, bacteria breached the mucosal barrier and caused bacteremia. Thus, as piglets in both infection groups showed disease symptoms and developed meningitis (Tables S4 and S5), it may be concluded that SsnA does not play a role in the pathogenesis. It is also conceivable that the disadvantage of the lack of nuclease can be compensated by the presence of other virulence factors.

Independently of the *S. suis* DNase activity, we detected NETs inside the CSF already in the early phase of infection (Figure 2) and were able to confirm findings from our previous study in the later phase of infection [2]. Neutrophils in CSF during the early phase of infection are able to release NETs containing PR-39 (Figure 2A,B). Human and murine NETs are stabilized against DNase degradation by the human cathelicidin LL-37 or the murine cathelicidin CRAMP [17,29]. In pigs PR-39 was also identified to protect DNA and NETs against degradation [2]. Here we showed in vivo that PR-39 in neutrophils is already present during transmigration through the choroid plexus epithelium in the early onset of meningitis. Furthermore, we confirmed a high amount of PR-39 in CSF as well as in neutrophils and NETs in the CSF compartment (Figure 2). For LL-37, a loss of its antimicrobial function against *Pseudomonas aeruginosa* was described when bound to DNA fibers [30]. Further studies are needed to clarify if the function of PR-39 in NET-structures is to kill microbes or only to prevent their spread by stabilizing NETs, or both. Furthermore, the function of other peptides such as PMAP-23 or PMAP-37, which are present in infected piglets (Figure S4), has not yet been investigated. 

As *S. suis* and neutrophils invade and infiltrate the meninges, we confirmed NET markers in situ by immunofluorescence staining in meninges of pigs with CNS disorders (Figure 3). However, it is remarkable that no NET fibers, as seen in CSF, were detectable in situ in the meninges, not even in pigs infected with the 10Δ*ssnA* strain. Based on our findings, we assumed that the host itself releases high amounts of DNases to destroy NETs and thereby reduces the possible tissue-damaging effect of NETs in the meninges [8,31]. Indeed, in the choroid plexus, DNase 1 was only found in animals with CNS disorders (Figure 4A). Interestingly, DNase was detectable in the choroid plexus in the area of transmigrating neutrophils. Furthermore, animals with meningitis showed high amounts of DNase 1 in CSF (Figure S9). A high DNase activity was also found in serum and in CSF independently of whether piglets were infected with *S. suis* 10 or 10Δ*ssnA* (Figure 4). In humans, DNase 1 alone is not able to destroy NETs completely; other host nucleases like DNase 1L3 and TREX1 are additionally needed [32]. The question of whether this finding also applies to pigs needs further investigation, but is still technically challenging for pigs based on lack of available tools.

Recently, an enhanced bacterial clearance in the CSF of *S. pneumoniae*-infected rats by increased antimicrobial activity of neutrophils when treated with DNase 1 was observed [11]. To further investigate the effect of DNase 1 on neutrophil activities against *S. suis*, we adapted a model of the blood–CSF barrier [23], with integrity during infection and transmigration of neutrophils (Figure S10). As no effect on neutrophil transmigration and killing of *S. suis* by adding DNase 1 was found in the classical model using cell culture medium and isolated neutrophils (Figure 5), we established a physiologically-improved blood–CSF barrier model with porcine CSF and whole blood in the respective compartments (Figure 6). To investigate if DNase 1 enhances the bacterial clearance in the CSF model, *S. suis* was directly applied with the same CFU/mL into the CSF. Therefore, *S. suis* was not transmigrating through the cell layer, and neutrophil transmigration was triggered with IL8. It is known that *S. suis* induces IL8 production in porcine choroid plexus epithelial cells [33], and an IL8 concentration that can be reached during meningitis [34] was added to the CSF compartment. In bacterial meningitis of children a significantly increased IL8 level was also described [35]. Efficient neutrophil transmigration was present in this optimized model, but the supplementation of DNase 1 did not cause any differences (Figure 7B). However, in the case of approximately 50% of the blood donors, an increased killing of *S. suis* 10 was detectable in the presence of DNase 1 (Figure 7D). Importantly, a statistical correlation between transmigrated neutrophils and bacterial killing was only confirmed when DNase 1 was added to the experimental setting (Figure 7F). Thus, these data lead to the assumption that DNase 1 does not alter the transmigration of neutrophils but might contribute to enhanced killing activity. These data are in line with the recently published phenotype that DNase 1 triggers antimicrobial activity of neutrophils during *Streptococcus pneumoniae* CNS infections in rats. The authors proposed that intact NETs are not beneficial during acute pneumococcal meningitis and that pneumococcal strains with low DNase-activity remain unaffected by NETs [11]. Treatment with DNase 1 released the bacteria from the NETs, and an increased level of neutrophil phagocytosis was found at the same time. In our study, an increased killing by neutrophils was only confirmed for the phagocytic sensitive capsule mutant of *S. suis* in the presence of DNase 1 and autologous plasma (Figure 8B). Without autologous plasma, the phenotype of increased killing changed in the other direction, assuming that NETs play a major role in the absence of plasma components and phagocytosis of *S. suis* is increased if NETs are degraded and opsonin factors are present. However, when using a capsulated *S. suis* 10 strain that exhibits high DNase activity, this phenomenon of DNase 1-mediated enhancement of neutrophil killing was not confirmed in our study and thus may depend highly on the bacterial strain and its virulence factors (Figure 7C,D). As the combination of plasma and DNase led to the best antimicrobial effect of neutrophils, this could also explain the difference between the two cell culture models used in our study. Although in the cell culture with purified neutrophils and the absence of plasma the killing effect was not influenced by DNase 1, an effect was detectable with whole blood. It is conceivable that next to neutrophils, plasma components of the whole blood can pass through the barrier into the CSF compartment in high amounts when barrier integrity is lost [36,37]. One possible factor is the complement factor C1q. The factor itself has no DNase activity, but it can highly increase serum DNase 1 activity [38]. Interestingly, it was found that complement factors and common complement pathway factors were increased in CSF of patients with bacterial meningitis, compared to controls [39].

As a natural host of *S. suis*, we used blood from male and female pigs with differing genetic backgrounds and of different ages, which were kept under common husbandry conditions. We thus hypothesize that the immune status of each individual pig might influence the results and explain the high variability among the individual pigs during cell culture experiments. In contrast to our study, the in vivo study with pneumococci was conducted with adult male Sprague Dawley rats, thus originating from a closed outbreed colony [11]. It is likely that they were kept under laboratory animal housing conditions, as usual, and therefore that all rats reacted in a similar fashion. Therefore, in future studies the influence of different blood donors should be investigated more in detail, in order to reflect the variable situations in conventional husbandry conditions. This might be transferred to the variable conditions in humans as the main reason for high inter-individual differences in innate immune reactions.

In summary, our results indicate that during *S. suis* meningitis the effects of host DNases seem to be more relevant than the effects of bacterial DNase. *S. suis* SsnA does not contribute to the pathogenesis of meningitis in piglets. However, host nucleases such as DNase 1 may participate in an efficient innate immune response by partially triggering the killing of *S. suis* by neutrophils when plasma is present. By immunofluorescence staining, we were able to show that DNase 1 is produced in the choroid plexus during acute meningitis (Figure 4A). Perhaps DNases are also stored in CSF for rapid deployment, eventually bound to actin to stay inactive until needed [40]. Whether treatment with DNase 1 is beneficial in *S. suis* meningitis, as discussed for pneumococcal meningitis [11] or used in cystic fibrosis [41,42,43], needs to be further investigated.

However, independently of nuclease production, both antimicrobial functions of neutrophils, phagocytosis, as well as NET-formation, are found in vivo in the CSF of infected piglets and may contribute to bacterial elimination. In our in vitro model *S. suis* was detectable extra- and intracellularly, as found in CSF of animals with CNS disorders (Figure S13). Interestingly, the production of “vital” NETs that are still able to be chemotactic active and phagocytize, are actually controversially discussed in the literature in comparison to “suicidal” NET release [44,45]. This phenomenon might explain our findings, as well as Mohanty’s findings that DNase treatment of neutrophils increases its antimicrobial functions by cleaving NET fibers and thereby supporting intracellular uptake of bacteria. Further studies should clarify the role of “vital” versus “suicidal” NETs in inhibiting the spread of *S. suis* in CSF and clarify the role of host DNases on neutrophil functions in greater detail to understand the underlying mechanisms.

## 4. Materials and Methods

### 4.1. Bacterial Strains and Growth Conditions

*Streptococcus suis* strain 10 (*S. suis* 10) is a virulent wild-type serotype 2 strain (*mrp ^+^*, *epf ^+^*, *sly ^+^*) of multilocus sequence type (ST) 1 and has been used for infection studies before [13,46]. The strain was isolated from a pig with pneumonia [24,47].

In addition to the wild-type strain, two isogenic knock-out mutants were used: *S. suis* strain 10∆*ssnA* (10∆*ssnA*), a nuclease A-deficient in-frame deletion mutant [10], and *S. suis* strain 10*cps*ΔEF (10*cps*ΔEF)*,* a capsule-deficient isogenic mutant [24].

Strains were cultivated out of frozen glycerol stocks on Columbia Agar with 7% Sheep Blood (Thermo Scientific™ PB5008A).

For the infection of piglets, bacterial strains were grown to early stationary phase in tryptic soy broth (TSB) without dextrose (Becton Dickinson, 286220, Franklin Lakes, NJ, USA) at 37 °C with 5% CO_2_. The 40 mL portions of the suspension were centrifuged and after discarding the supernatant bacteria were suspended in 3 mL PBS, leading to a bacterial concentration of 4 × 10^9^ CFU/mL. The exact CFU/mL was determined by plating serial dilutions on blood agar plates (Columbia Agar with 7% Sheep Blood; Thermo Scientific™ PB5008A, Waltham, MA, USA).

For in vitro assays, working cryostocks of bacterial strains were generated by growing the bacterial strains to early stationary phase in TSB without dextrose (Becton Dickinson, 286220, Franklin Lakes, NJ, USA) at 37 °C with 5% CO_2_. Aliquots with 15% Glycerol (Sigma Aldrich, 13487-2, St. Louis, MO, USA) were frozen in liquid nitrogen and stored at −80 °C.

### 4.2. Experimental Infection of Piglets

All pigs used in this study were castrated male German Landrace piglets from a herd known to be free of *S. suis* 10 but not free of *S. suis* in general. Handling and treatment of animals was in strict accordance with the principles of the European Convention for the Protection of Vertebrate Animals Used for Experimental and Other Scientific Purposes, as well as the German Animal Protection Law. The animal experiments were approved by the Committee on Animal Experiments of the Lower Saxonian State Office for Consumer Protection and Food Safety (Niedersächsisches Landesamt für Verbraucherschutz und Lebensmittelsicherheit) under permit numbers 33.14-42502-04-12/0965 (last update 2013) and 33.12-42502-04-12/0991 (last update 10/2017). Post-infection, animals were monitored every eight hours for internal body temperature, food uptake, behavior, breathing, posture, motion sequence and lameness.

### 4.3. Survival Experiment

Eighteen 4–5-week-old pigs were divided into two groups. After pre-disposition with 1% acetic acid under anesthesia (azaperon 2 mg/kg body weight (Stresnil, Elanco GmbH, Cuxhaven, Germany), ketamine 10 mg/kg bodyweight (Ursotamin, Serumwerke Bernburg, Bernburg, Germany)), the animals in the respective groups were intranasally challenged with 1 × 10^9^ CFU *S. suis* 10 or 10Δ*ssnA*, as previous described [13]. The health status of the animals was monitored every 8 h. A piglet was classified as morbid if a body temperature of ≥40.2 °C or/and severe clinical signs of an acute disease were observed. In case of high fever (≥40.5 °C), apathy and anorexia persisting over 36 h, as well as in all cases of central nervous system dysfunction or clinical signs of acute polyarthritis, animals were euthanized for reasons of animal welfare. All surviving piglets were sacrificed 13 days post-infection (dpi). Histological samples for Figure 3A were taken from the same pigs as the already-published CSF samples [2]. All information about the experiment conducted under permission number 33.14-42502-04-12/0965 was published elsewhere [14].

### 4.4. Early Phase Experiment

Thirty-nine 8-week-old piglets were divided into three groups and infected with 6 × 10^9^ CFU *S. suis* 10, 10Δ*ssnA* or mock infected with PBS (uninfected) as described above. 

### 4.5. Sample Collection

During anesthesia, before infection, swabs of the tonsils, heparin blood and serum samples were collected. Piglets in the survival experiment were sacrificed as described above, 13 days post-infection at the latest. In the early phase experiment, we sacrificed the first half of each group (uninfected 5/11, infected 7/14) 48 h post-infection, the second half of all animals (uninfected 6/11, infected 6/14) 96 h post-infection. One animal of the *S. suis* Δ*ssnA* infected group had to be sacrificed 72 h after infection and one animal of the *S. suis* 10 infected group 86 h after infection due to the clinical symptoms of meningitis for animal welfare reasons.

In both experiments the animals were anesthetized for euthanasia as already described and heparin blood and serum samples were collected. After euthanasia with T61 i.v., CSF samples were taken by puncturing of the cisterna magna [48]. During section swabs of the brain surface, the mitral valve, the pleura, the pericard and the peritoneum were taken for bacteriology. In addition, for bacteriology and histology, organ samples of the brain, liver, spleen, tonsils, heart, lung, as well as carpal and tarsal joints, were collected. Samples for histology were stored immediately in 10% buffered formalin.

### 4.6. Re-Isolation of Infection Strains

Organ and swab samples collected during section of the animals were spread onto blood agar plates, with tonsils additionally on selective agar for *Streptococci* (Oxoid, PB5049A, Waltham, MA, USA). Isolated *S. suis* strains were identified by matrix-assisted laser desorption ionization time-of-flight mass spectrometry (MALDI-TOF MS). Isolation of the challenge strain was confirmed by multiplex PCR detecting *mrp, epf, sly, arcA, gdh, cps1, cps2, cps7* and *cps9* [49], as well as PCR amplifying the *ssnA* gene, to verify the knock-out in the respective isolates. Primers used for *ssnA* detection are listed in Table S7 and cycling consisted of 30-times denaturation at 94 °C for 1 min, annealing at 58 °C for 45 s, elongation at 68 °C for 5 min and final elongation at 68 °C for 5 min.

### 4.7. Blood Survival Assay

Survival in the blood was determined by infecting 0.5 mL porcine whole blood with 3 × 10^5^ CFU either *S. suis* 10 or 10∆*ssnA* for 2 h. During infection, the samples were rotated on a rotator at 8 rounds per minute in an incubator at 37 °C. At time-points 0 h and 2 h the CFU was determined by plating serial dilutions on blood agar plates (Thermo Scientific™ PB5008A, Waltham, MA, USA). The survival factor was calculated by dividing the number of CFUs after 2 h of incubation by the number of CFUs at 0 h. 

### 4.8. Pico Green Quantification Assay

A Pico Green quantification assay (Quant-iT™ PicoGreen^®^; Invitrogen, Carlsbad, CA, USA) was performed to determine free DNA in CSF and serum, as described previously [12].

### 4.9. Histology of the Brain

Immediately after extraction, the entire brain was fixed in 10% formalin (buffered) for a maximum of 72 h. The regions of the corpus striatum, hippocampus and cerebellum, the latter two containing the choroid plexus, were embedded in paraffin and cut into 2–4 µm sections for hematoxylin-eosin (HE) staining and immunofluorescence staining. 

### 4.10. Cytospin of CSF

At a maximum of 30 min after collection of CSF, 100 µL CSF of all non-infected animals and of animals without fever or altered CSF were transferred to CellView^®^ slides (Greiner bio-one 543079, Kremsmünster, Austria). From all animals with CNS disorders, fever or altered CSF, cells in CSF were counted. The cell number was adjusted with HBSS to 1 × 10^4^ to 1 × 10^5^ cells/100 µL and cells were added to the CellView^®^ slides. The slides were centrifuged at 370 g for 5 min at room temperature and fixed with a final concentration of 4% paraformaldehyde (PFA).

### 4.11. Staining and Microscopy

#### 4.11.1. Staining of CSF in CellView^®^ Slides

- Staining of NETs and *S. suis*: CSF was fixed with 4% paraformaldehyde (Science Services, E15710-25, Munich, Germany). Staining was performed as previously described [2] using as first antibodies mouse IgG2a anti-DNA/histone (Millipore MAB3864; 0.55 mg; 1:1000, Billerica, MA, USA) and rabbit IgG anti-PR39 (1:75) or rabbit IgG anti-*S. suis* [19] (1:500). After washing, the DNA was stained with aqueous Hoechst 33,342 (Stock 50mg/mL, Sigma B-2261, St. Louis, MO, USA) and covered with ProLong™ Gold Antifade Mountant (Invitrogen, P36930, Carlsbad, CA, USA). The samples were examined microscopically on a Leica TCS SP5 AOBS confocal inverted-base fluorescence microscope with a HCX PL APO 40 × 0.75–1.25 oil immersion objective with an Argon, 405 nm and 633 nm laser. The settings were adjusted using isotype control antibodies in separate preparations.
- Staining of NETs and intra- and extracellular *S. suis*: staining was performed as previously described [2] using first antibodies mouse IgG2a anti-DNA/histone (Millipore MAB3864, Billerica, MA, USA; 0.55 mg; 1:1000) and rabbit IgG anti-*S. suis* [19] (1:500). After washing the DNA was stained with aqueous Hoechst 33,342 [(Stock 50 mg/mL, Sigma B-2261, St. Louis, MO, USA) and covered with ProLong™ Gold Antifade Mountant (Invitrogen, P36930, Carlsbad, CA, USA). The samples were examined microscopically on a Leica TCS SP5 AOBS confocal inverted-base fluorescence microscope with a HCX PL APO 40 × 0.75–1.25 oil immersion objective with an Argon, 405 nm, 561 nm and 633 nm laser. The settings were adjusted using isotype control antibodies in separate preparations.

#### 4.11.2. Staining of Slides

Slides of cell culture experiments and with isolated neutrophils: the co-staining of NETs and elastase, as well as the extracellular and intracellular *S. suis* staining, were performed as previously described [2].

#### 4.11.3. Staining of 2–4 µm Paraffin Embedded Brain Sections

- Hematoxylin-Eosin (HE) Staining: Automated dying in LEICA ST 4040 with 0.1 % hematoxylin (Roth, Karlsruhe, Germany) and 1 % eosin (Roth, Karlsruhe, Germany).
- Immunofluorescence Staining: The staining was performed as previously described [12] with the following changes. First antibodies mouse IgG2a anti-DNA/histone (Millipore MAB3864, Billerica, MA, USA; 0.55 mg; 1:100) or mouse anti-PR-39 (Lionex, Braunschweig, Germany; 1:100) and rabbit anti-elastase (Abcam, ab1876, Cambridge, UK; 1:50) or rabbit IgG anti-*S. suis* [19] (1:500) or rabbit anti-DNase 1 (Invitrogen; PA5-22017, Carlsbad, CA, USA; 1:100) dissolved in blocking buffer were incubated overnight 4 °C, while gently shaking. The samples were examined microscopically on a Leica TCS SP5 AOBS confocal inverted-base fluorescence microscope with HCX PL APO 40 × 0.75–1.25 and HCX PL APO lambda blue 63 × 1.40 oil immersion objectives with an Argon, 405 nm and 633 nm laser. The settings were adjusted using isotype control antibodies in separate preparations.

### 4.12. DNase Activity Test

To 50 µL serum or CSF of each pig, 1 µg deoxyribonucleic acid sodium salt from calf thymus (Sigma, D3664, St. Louis, MO, USA) was added. The samples were incubated at 37 °C for 20 h. After incubation, samples were run on an 1% electrophoresis gel containing Roti^®^-GelStain (Roth, 3865.1, Karlsruhe, Germany) at 100 V for 30 min. Bands were detected with Biorad ChemiDoc MP (Hercules, California) and the degradation of the calf thymus DNA was classified into 4 groups between no (0) and total (3) degradation, by eye (Figure S8D).

### 4.13. DNase 1, PR-39, PMAP-23, PMAP-37 ELISA

DNASE 1 ELISA Kit (pig) (Aviva Systems Biolo, USAgy OKEH03902, San Diego, CA, USA), PR-39 ELISA Kit (MBS9428782, San Diego, CA, USA), PMAP-23 ELISA Kit (OKWB00337, San Diego, CA, USA) and PMAP-37 ELISA Kit (ELI-37324p, San Diego, CA, USA) were used to analyze CSF samples. The tests were performed following the manufacturers’ instructions.

### 4.14. Cell Culture

Porcine choroid plexus epithelial cells (PCP-R) [50] were seeded on the underside of filter inserts with 3 µm pores (Greiner bio-one, 662631, Kremsmünster, Austria) and transmigration assays were performed as previously described [23], with the following modifications.

- Cell Culture Model of the Porcine Blood–CSF Barrier (Figure 5A): After three days’ incubation, transepithelial electrical resistance (TEER) was measured with a Millicell^®^ ERS-2 (Millipore, Billerica, MA, USA) voltmeter to control the barrier density. Some filters were infected with *S. suis* 10 for one hour in the blood compartment. Before adding the freshly isolated porcine neutrophils, medium in the blood compartment was changed in all filters. Transmigration of neutrophils was performed for 4 h at 37 °C and 5% CO_2_. Serial dilutions of transmigrated bacteria were plated on blood agar (Columbia Agar with 7% Sheep Blood; Thermo Scientific™ PB5008A, Waltham, MA, USA) to determine the CFU/mL and 250 µL medium of the CSF compartment was fixed with a final concentration of 4% PFA (Paraformaldehyde 16% Science Services E15710, Munich, Germany) for flow cytometric analysis. TEER was measured again. The experiment was repeated three times on independent days.
- Cell Culture Model of the Porcine Blood–CSF Barrier Mimicking Physiological Conditions (Figure 6): Filters were transferred in new wells with 990 µL pooled CSF of healthy piglets (euthanasia of these pigs was approved and registered by the local Animal Welfare Officer in accordance with the German Animal Welfare Law under number TiHo-T-2019-14, last update 07/2019). To attract neutrophils, 50 ng interleukin 8 (IL8) (Recombinant Porcine IL-8/CXCL8, R&D Systems 535-IN-025, Minneapolis, MN, USA) was added to all wells. To half of the wells, 20 U DNase 1 (Serva 18535.02) was added. CSF of half of the wells was infected with 1–2 × 10^2^ CFU *S. suis* 10. Thus ultimately yielded: non-infected wells, non-infected wells with DNase 1, infected wells and infected wells with DNase 1. In the upper compartment, 400 µL heparinized whole pig blood (animal permit registered at the Lower Saxonian State Office for Consumer Protection and Food Safety (Niedersächsisches Landesamt für Verbraucherschutz und Lebensmittelsicherheit) under no. 33.9-42502-05-18A302, last update 07/2018) was added and incubated at 37 °C and 5% CO_2_ for 4 h. After incubation, serial dilutions of CSF were plated on blood agar plates (Columbia Agar with 7% Sheep Blood; Thermo Scientific™ PB5008A, Waltham, MA, USA) to determine the CFU/mL, and 250 µL CSF was fixed with a final concentration of 4% PFA (Paraformaldehyde 16% Science Services E15710, Munich, Germany) for flow cytometric analysis. TEER was measured again. The experiment was repeated five times on independent days. CSF was collected from healthy euthanized pigs. The euthanasia of these pigs was approved and registered by the local Animal Welfare Officer in accordance with the German Animal Welfare Law under number TiHo-T-2019-14.

### 4.15. Flow Cytometry

Two hundred microliters of PFA-fixed CSF samples from the cell culture experiments were measured in an Attune NxT Flow Cytometer (Invitrogen, Carlsbad, CA, USA) with forward and sideward scatter using a 488 nm laser. Data were analyzed with FlowJo^TM^ version 10.6.1 (Ashland, OR, USA) software by drawing a gate to exclude the debris.

### 4.16. Neutrophil Isolation

Porcine neutrophil granulocytes were isolated from fresh heparinized blood (animal permit registered at the Lower Saxonian State Office for Consumer Protection and Food Safety (Niedersächsisches Landesamt für Verbraucherschutz und Lebensmittelsicherheit) no. 33.9-42502-05-18A302) by density gradient centrifugation using Biocoll ^TM^ separating solution 6115 (Biochrome, L 6115, Berlin, Germany), as described previously [23]. After removal of the erythrocytes by hypotonic lysis, PMNs were suspended in RPMI 1640 medium (ThermoFisher, 11835063, Waltham, MA, USA) or cell culture medium.

### 4.17. Neutrophil Killing Assay

In total, 2 × 10^5^ freshly isolated porcine neutrophils in RPMI were infected with 2 × 10^5^ CFU 10*cps*ΔEF [24] (MOI 1) in a total volume of 190 µL in a 48-well plate (Greiner Bio-One, 677102, Kremsmünster, Austria). Ten percent plasma of the blood donation pig and 5 U DNase 1 (Serva 18535.02) were added, so we tested samples with (a) bacteria and neutrophils, (b) bacteria, neutrophils and Dnase 1, (c) bacteria, neutrophils and plasma and (d) bacteria, neutrophils, plasma and Dnase 1. At time-points 0 h and 2 h of incubation at 37 °C, 5% CO_2_ serial dilutions were plated on blood agar plates (Columbia Agar with 7% Sheep Blood; Thermo Scientific™ PB5008A) to determine the CFU/mL.

### 4.18. Statistical Analysis

Data were analyzed using Excel 2010 and 2016 (Microsoft, Albuquerque, NM, USA). Statistical analyses were performed with GraphPad Prism Version 8.0.1 (San Diego, CA, USA). A detailed description of tests used can be found in the figure legends, but the *p*-value has the same settings in all graphs (* *p* < 0.05, ** *p* < 0.01, *** *p* < 0.001, **** *p* < 0.0001).

## Supplementary Materials

Supplementary Materials can be found at https://www.mdpi.com/1422-0067/21/15/5289/s1, Table S1: Assessment of morbidity and mortality after intranasal infection in the survival experiment; Table S2: Scoring of fibrinosuppurative lesions of piglets in the survival experiment; Table S3: Re-isolation of the infection strains after intranasal infection of piglets in the survival experiment; Table S4: Assessment of morbidity after intranasal infection in the early phase experiment; Table S5: Scoring of brain lesions of piglets in the early phase experiment; Table S6: Re-isolation of the infection strains after intranasal infection of piglets in the early phase experiment; Table S7: Primers used for *ssnA* detection; Figure S1: No differences in survival of *S. suis* 10 and 10Δ*ssnA* in blood of piglets before infection; Figure S2: Development of body temperature in the early phase experiment; Figure S3: Free DNA, as a marker for NETs, increases in CSF of animals with clinical meningitis but not in serum post-infection; Figure S4: ELISA of porcine antimicrobial peptides PMAP-23 and PMAP-37 in CSF; Figure S5: Regions of sagittal cuts from porcine brain for histology analysis; Figure S6: Single channels of immunofluorescence staining from Figure 3B; Figure S7: Single channels of immunofluorescence staining from Figure 4A; Figure S8: Gel electrophoresis gels of the DNase activity assay (Figure 4C); Figure S9: Host nuclease DNase1 is detectable in CSF during *S. suis* meningitis; Figure S10: The barrier integrity of PCP-R cells was tight during *S. suis* 10 infection and neutrophil transmigration; Figure S11: Impact of IL8 on transmigration of isolated porcine neutrophils through a cell layer of porcine choroid plexus epithelial cells; Figure S12: The barrier integrity of PCP-R cells was tight during *S. suis* 10 infection and neutrophil transmigration in the more physiological cell culture system; Figure S13: More *S. suis* are found extracellularly than intracellularly in infected CSF of piglets with clinical meningitis.
