# Supplementary material for: Role of Bacterial and Host DNases on Host-Pathogen Interaction during Streptococcus suis Meningitis
Source: Int J Mol Sci. 2020 Jul 25;21(15):5289. doi: 10.3390/ijms21155289 (PMC7432635; doi:10.3390/ijms21155289)
Supplement: Supplementary file 1 [file ijms-21-05289-s001.pdf]

Supplemental table 1 **Assessment of morbidity and mortality after intranasal infection in the survival experiment.**

age of infected pigs (wk)	total no. of infected pigs	*S. suis* strain	no. of pigs/total no. of pigs [a]				
			morbidity	mortality	severe clinical symptoms[b]	maximum body temp (°C)	
						≤40.2	≥40,8
4-5	9	10	5/9	3/9	3/9	4/9	5/9
4-5	9	10ΔssnA	2/9	1/9	2/9	9/9	0/9

[a] German Landrace piglets from a herd free of *sly*+ *epf*+ *mrp*+ *cps9 S. suis* strains.

[b] In particular severe depression, apathy, persistent anorexia, acute severe lameness and/or neural disorder.

Supplemental table 2 **Scoring of fibrinosuppurative lesions of piglets in the survival experiment.**

age of infected pigs (wk)	total no. of infected pigs	*S. suis* strain	no. of pigs / total no. of pigs															ω [e]
			brain			serosae			joint			spleen and liver			lung			
			meningitis, choroiditis			pleuritis or peritonitis			synovialitis			splenitis [a] or hepatitis			pneumonia			
			5 [b]	3 [c]	1 [d]	4 [b]	2 [c]	1 [d]	4 [b]	2 [c]	1 [d]	4 [b]	2 [c]	1 [d]	4 [b]	2 [c]	1 [d]	
4-5	9	10	1/9	2/9*	1/9**	0/9	0/9	0/9	1/9	1/9	1/9	4/9	4/9	0/9	2/9	1/9	1/9	3
4-5	9	10ΔssnA	0/9	0/9	0/9	2/9	0/9	0/9	2/9	0/9	0/9	6/9	2/9	1/9	3/9	0/9	0/9	3,33

* minimally focal/multifocal lymphoplasmacellular Plexus chorioiditis (not in score)

** minimally focal purulent Plexus chorioiditis (not in score)

[a] Neutrophilic accumulation of the splenic red pulp.

[b] Scoring of 4 and 5 indicates moderate to severe diffuse or multifocal fibrinosuppurative inflammations.

[c] Scoring of 2 and 3 indicates mild focal fibrinosuppurative inflammation

[d] Individual single perivascular neutrophils received a score of 1.

[e] $\omega = \Sigma score_{max}/n_{animals}$ (Baums et al., 2006).

Supplemental table 3 **Re-isolation of the infection strains after intranasal infection of piglets in the survival experiment.**

S. suis strain	Number of piglets positive for the isolation of the challenge strain in an inner organ[b], serosa or joint fluid	number of piglets from total in which the *S. suis* challenge strain[a] was isolated from							
		tonsils	lung[c]	serosa[d]	spleen	liver	brain, CSF[e]	joint fluid[f]	endocard
10	2/9	5/9	0/9	0/9	0/9	0/9	2/9	1/9	0/9
10Δ*ssnA*	2/9	5/9	1/9	0/9	0/9	0/9	0/9	1/9	0/9

[a] The challenge strain was identified through PCR.

[b] Inner organ refers to lung, spleen, liver, brain, CSF or endocard but not the tonsils.

[c] One cranial lobe was investigated.

[d] Pleural, peritoneal or pericardial cavity.

[e] Cerebrospinal fluid.

[f] Punctures of both tarsal and carpal joints were investigated in each animal. In case of lameness additional joint punctures of the respective limb were screened.

Supplemental table 4 **Assessment of morbidity after intranasal infection in the early phase experiment.**

age of infected pigs a (wk)	total no. of infected pigs	*S. suis* strain	no. of pigs / total no. of pigs [a]				
			morbidity	severe clinical symptoms[b]	maximum body temp (°C)		
					≤40.2	40.3 - 40.7	≥40.8
8	14	10	4/14	1/14	7/14	3/14	4/14
8	14	10Δ*ssnA*	4/14	2/14	9/14	1/14	4/14
8	11	control	0/11	0/11	9/11	2/11	0/11

[a] German Landrace piglets from a herd free of *S. suis* 10.

[b] in particular severe depression, apathy, persistent anorexia, acute severe lameness and/or neural disorder.

Supplemental table 5 **Scoring of brain lesions of piglets in the early phase experiment**

S. suis strain	isolation infection strain all organs	isolation infection strain brain or CSF	no. of pigs / total no. of pigs									ω [f]
			brain									
			meningitis			plexus choroiditis			encephalitis			
			5 [b]	3 [c]	1 [d]	4 [b]	3 [c]	2 [d]	4 [b]	2 [c]	1 [d]	
10	7/14	1/14	1/14	0/14	0/14	1/14	0/14	1/14	0/14	0/14	0/14	0,5
10Δ*ssnA*	6/14	3/14	1/14	1/14	1/14	0/14	2/14	0/14	0/14	0/14	0/14	0,64
control	0/11	0/11	0/11	0/11	0/11	0/11	0/11	0/11	0/11	0/11	0/11	0

[b] scoring of 4 and 5 indicates moderate to severe diffuse or multifocal fibrinosuppurative inflammations.

[c] scoring of 2 and 3 indicates mild focal fibrinosuppurative inflammation

[d] individual single perivascular neutrophils received a score of 1.

[e] $\omega = \Sigma score_{max}/n_{animals}$ (Baums et al., 2006).

Supplemental table 6 **Re-isolation of the infection strains after intranasal infection of piglets in the early phase experiment.**

S. suis strain	number of piglets positive for the isolation of the challenge strain in an inner organ[b], serosa or joint fluid	number of piglets in which the *S. suis* challenge strain[a] was isolated from							
		tonsils	lung [c]	serosa [d]	spleen	liver	brain, CSF [e]	joint fluid [f]	endocard
10	4/14	4/14	2/14	1/14	0/14	2/14	1/14	0/14	1/14
10Δ*ssnA*	5/14	2/14	1/14	0/14	3/14	4/14	3/14	1/14	2/14
control	0/11	0/11	0/11	0/11	0/11	0/11	0/11	0/11	0/11

[a] the challenge strain was identified through PCR.

[b] inner organ refers to lung, spleen, liver, brain, CSF or endocard but not the tonsils.

[c] one cranial lobe was investigated.

[d] pleural, peritoneal or pericardial cavity.

[e] cerebrospinal fluid.

[f] punctures of both tarsal and carpal joints were investigated in each animal.
In case of lameness, additional joint punctures of the respective limb were screened.

Tab. S7 Primers used for *ssnA* detection. Cycling consisted of 30 times denaturation at 94 °C for 1 min, annealing at 58 °C for 45 s, elongation at 68 °C for 5 min and final elongation at 68 °C for 5 min.

Primer pair	Sequence 5'-3'
praeSsnASacI	TCAAGAGCTCTGGGAGACAGTTTGAGG
postSsnaSphI	CCAGCATGCAAGGGTGTCATGGAGTTGT

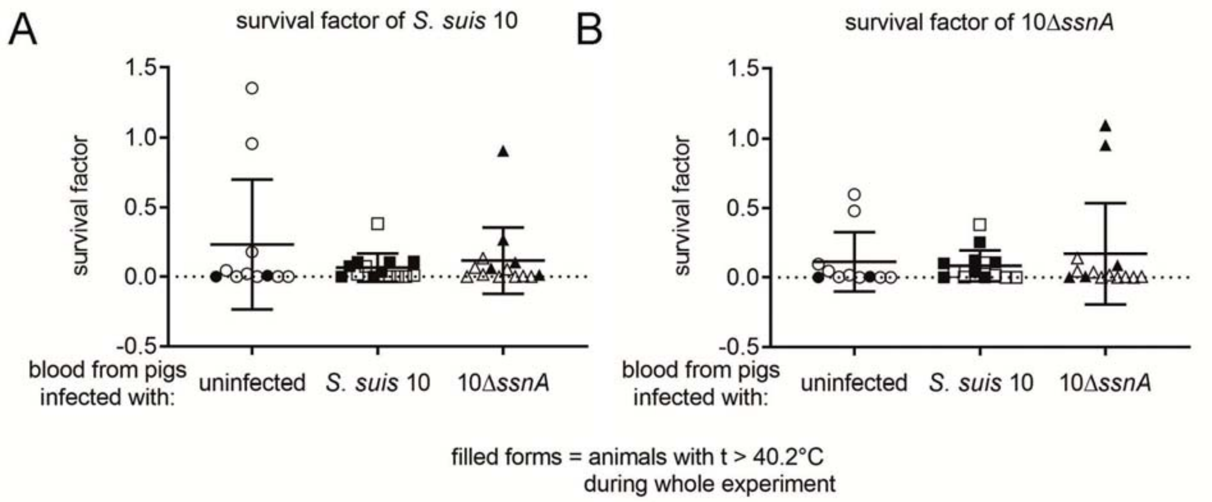

Fig. S1: No differences in survival of *S.suis*10 and 10Δ*ssnA* in blood of piglets before infection.

In a bactericidal assay we infected *in vitro* blood of all piglets with both infection strains pre-animal experiment (A) analysis with *S.suis*10, B) analysis with 10Δ*ssnA*). No differences in bactericidal effect of blood of animals in all groups to *S.suis*10 or 10Δ*ssnA* were detected. In almost all animals very low survival factors for both bacterial strains were found. The survival factor was higher than one only for one animal in the control group infected with *S. suis* 10 (A) and one animal in the 10Δ*ssnA* group infected with 10Δ*ssnA* (B). The survival factor was calculated by dividing the CFU/ml 2h by the CFU/ml 0h. Data shown as mean ± SD. Statistical analysis: one-way ANOVA.

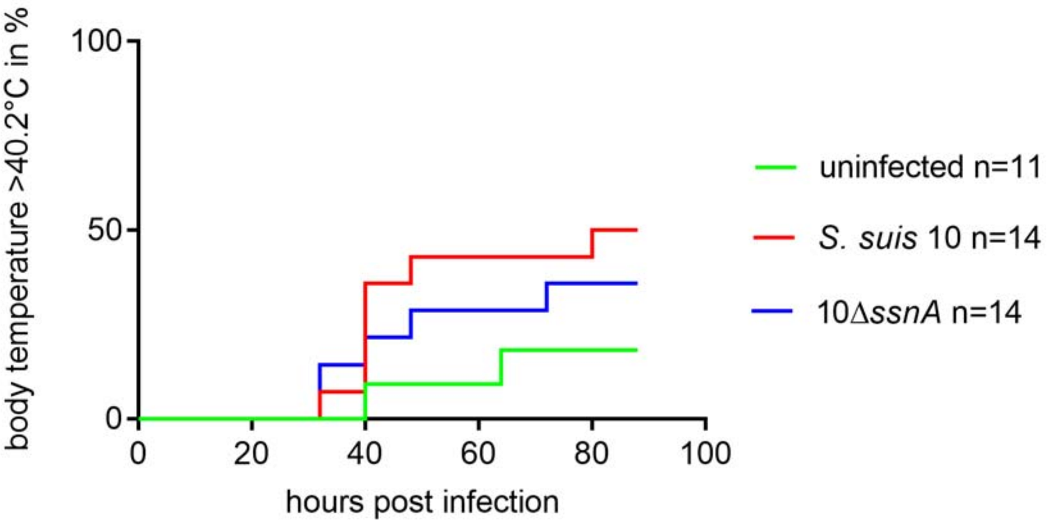

Fig. S2 Development of body temperature in the early phase experiment.

Slightly more animals of the *S.suis*10 infected group showed higher body temperatures than animals of the 10Δ*ssnA* infected group.

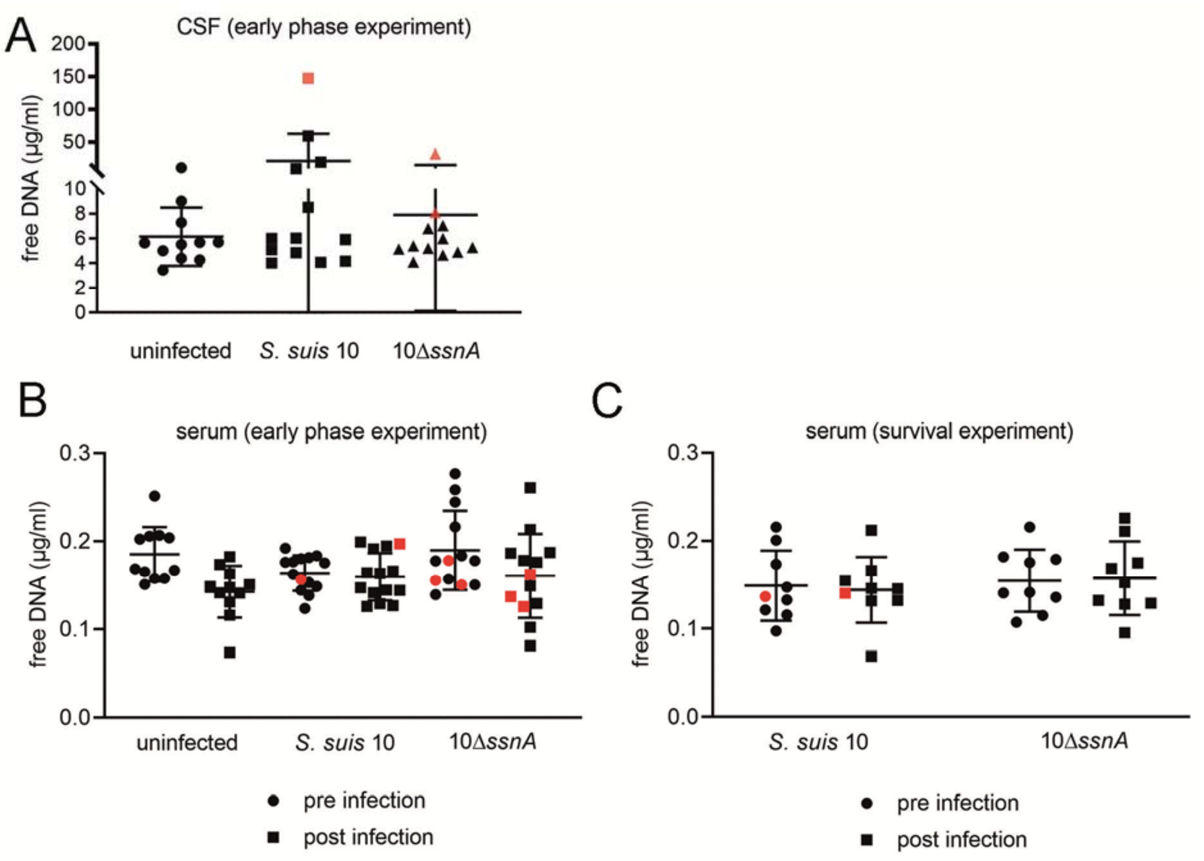

Fig. S3 Free DNA, as a marker for NETs, increases in CSF of animals with clinical meningitis but not in serum post infection.

The amount of free DNA was analyzed with Pico Green quantification assay A) In CSF of animals of the early phase experiment the piglet with clinical meningitis, infected with *S.suis*10, showed the highest amount of free DNA. In the group infected with 10Δ*ssnA* also one of the animals with clinical meningitis had the highest amount of free DNA in CSF, but lower than an animal without clinical meningitis of the *S. suis* 10-infected group. B and C) Free DNA was analyzed in serum pre and post infection from pigs out of both animal experiments. No differences were detected between the infection groups before and after the infection. Animals with clinical meningitis (red marks) do not show high free DNA in the serum. Data shown as mean ± SD. Statistical analysis: one-way ANOVA.

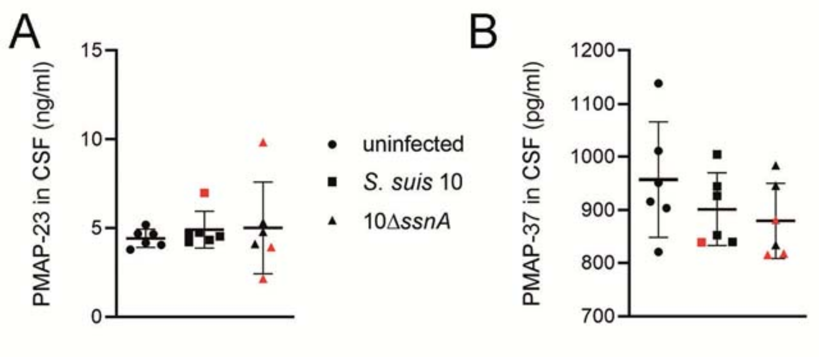

Fig. S4 ELISA of porcine antimicrobial peptides PMAP-23 and PMAP-37 in CSF. Neither for PMAP-23 (A) nor for PMAP-37 (B) differences in the amount inside CSF was detectable between all three infection groups from the early phase experiment. Animals with clinical meningitis (red marks) show variable results for PMAP-23 and the amount of PMAP-37 in these animals is low. Data shown as mean ± SD. Statistical analysis: unpaired Mann-Whitney test.

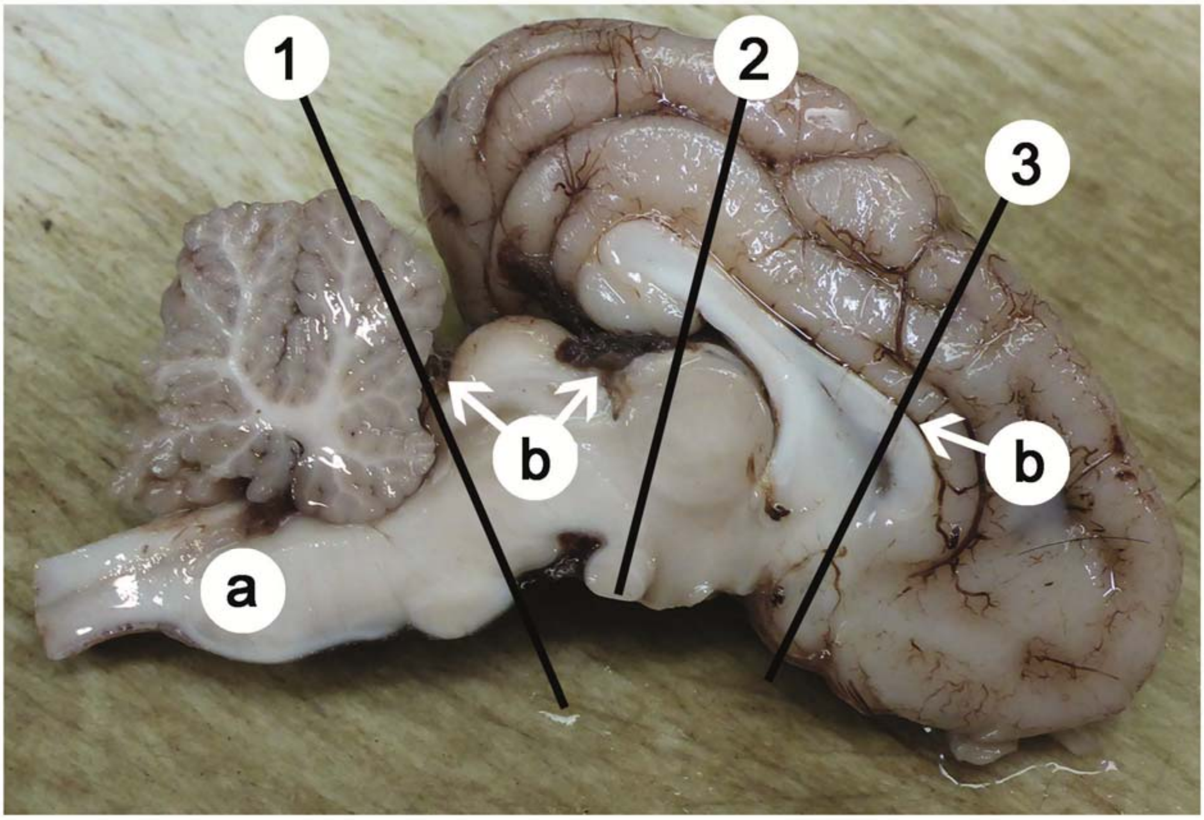

Fig. S5 Regions of sagittal cuts off porcine brain for histology analysis. a) medulla oblongata, b) ventricles with choroid plexus, the black lines mark regions were samples for histology are taken: 1) area cerebellum, 2) area hippocampus and 3) area corpus striatum. .

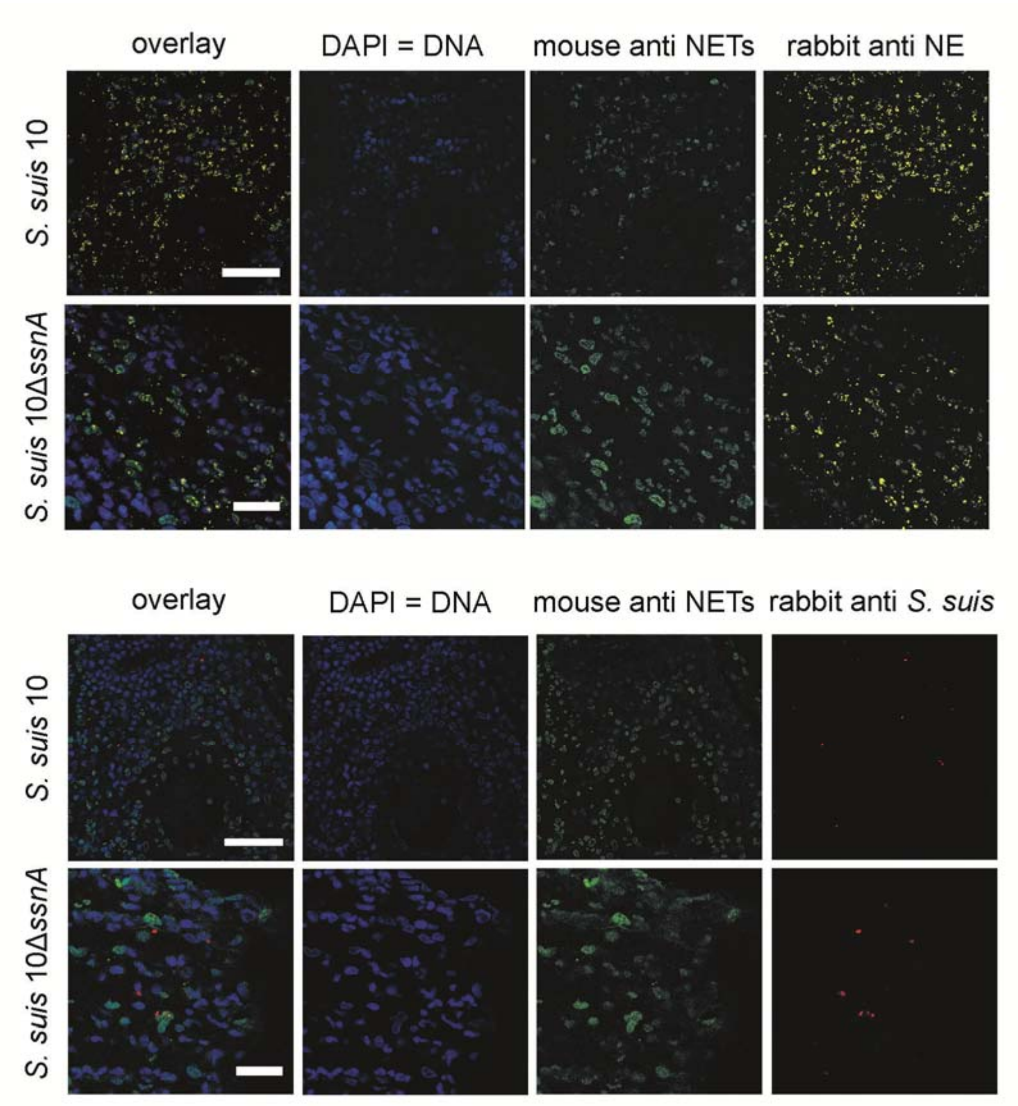

Fig. S6 Single channels of immunofluorescence staining from Fig. 3B (blue =DNA (DAPI), green=DNA/ histone-1-complexes (NETs), red = *S.suis*, yellow = elastase (NE), (scale bar = *S.suis*10 = 50 µm, *S.suis* 10Δ*ssnA* = 20µm).

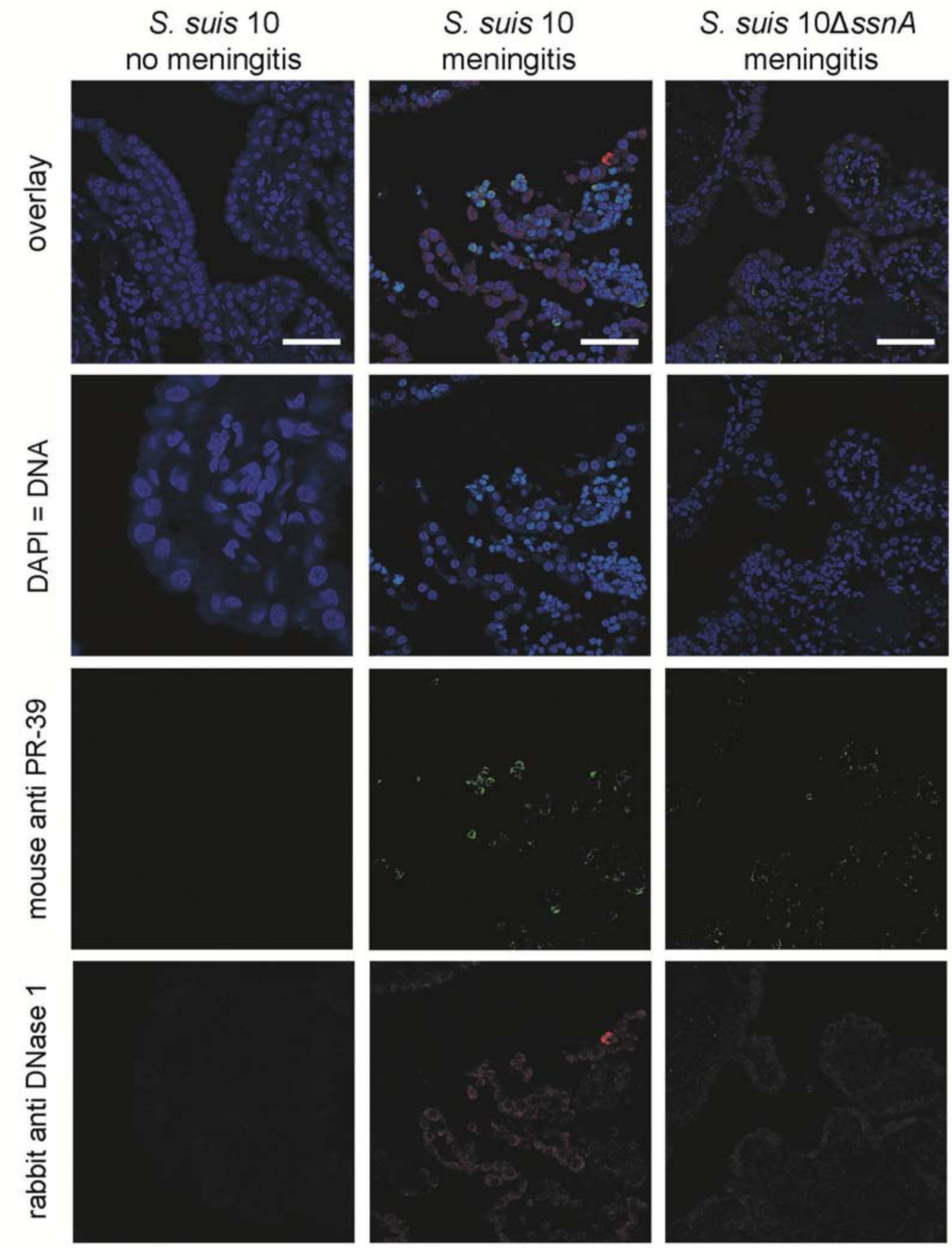

Fig. S7 Single channels of immunofluorescence staining from Fig. 4A (blue =DNA (Hoechst), green= PR-39, red = DNase1. (scale bar 50 µm).

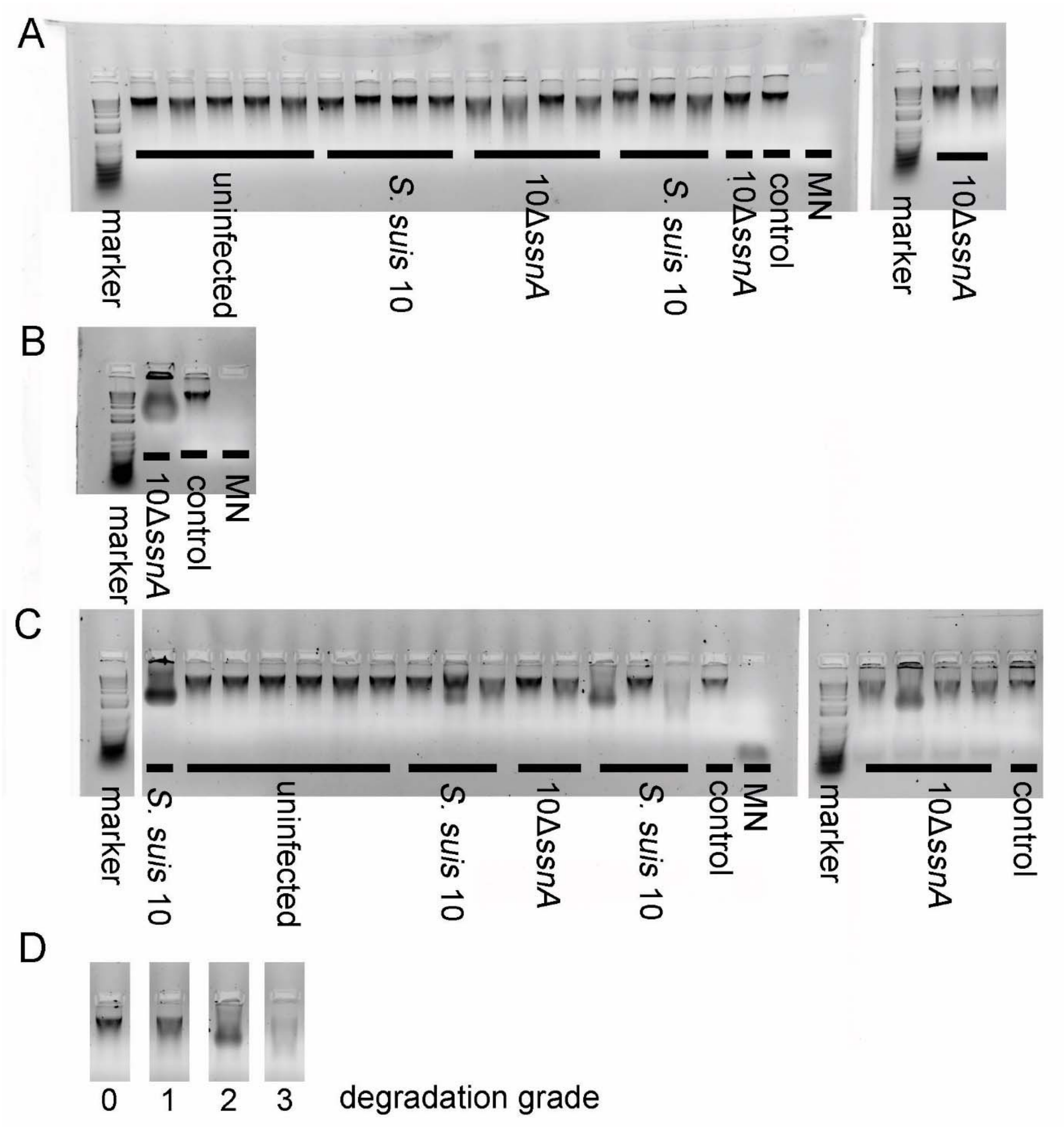

Fig. S8 Gel electrophoresis gels of the DNase activity assay (Fig.4c).

Ex vivo degradation of calf thymus DNA by nucleases of the CSF after 20 h incubation in CSF at 37 °C and 5 % CO_2 on a 1 % agarose gel. Each well shows one individual tested animal. A) Samples from 48h post infection, B), Samples from 72h post infection, C) Samples from 96h post infection. D) The selected examples show the evaluation of the 4 different degradation grades. MN= micrococcal nuclease was used as positive degradation control of DNA, in control only PBS was used.

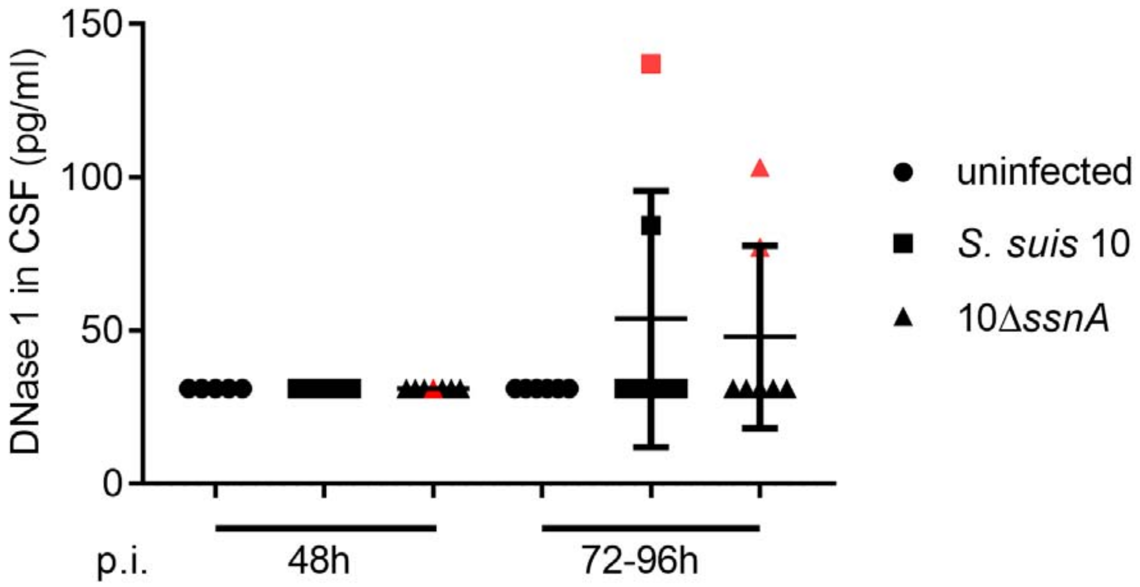

Fig. S9 Host nuclease DNase1 is detectable in CSF during *S.suis* meningitis.

A high amount of host nuclease DNase1 was found in CSF of animals with clinical meningitis (red marks) by ELISA 96h post infection. Unfortunately, no differentiated statements are possible for all animals, as most of the samples were below the detection limit. Data shown as mean ± SD. Statistical analysis: unpaired Mann-Whitney test.

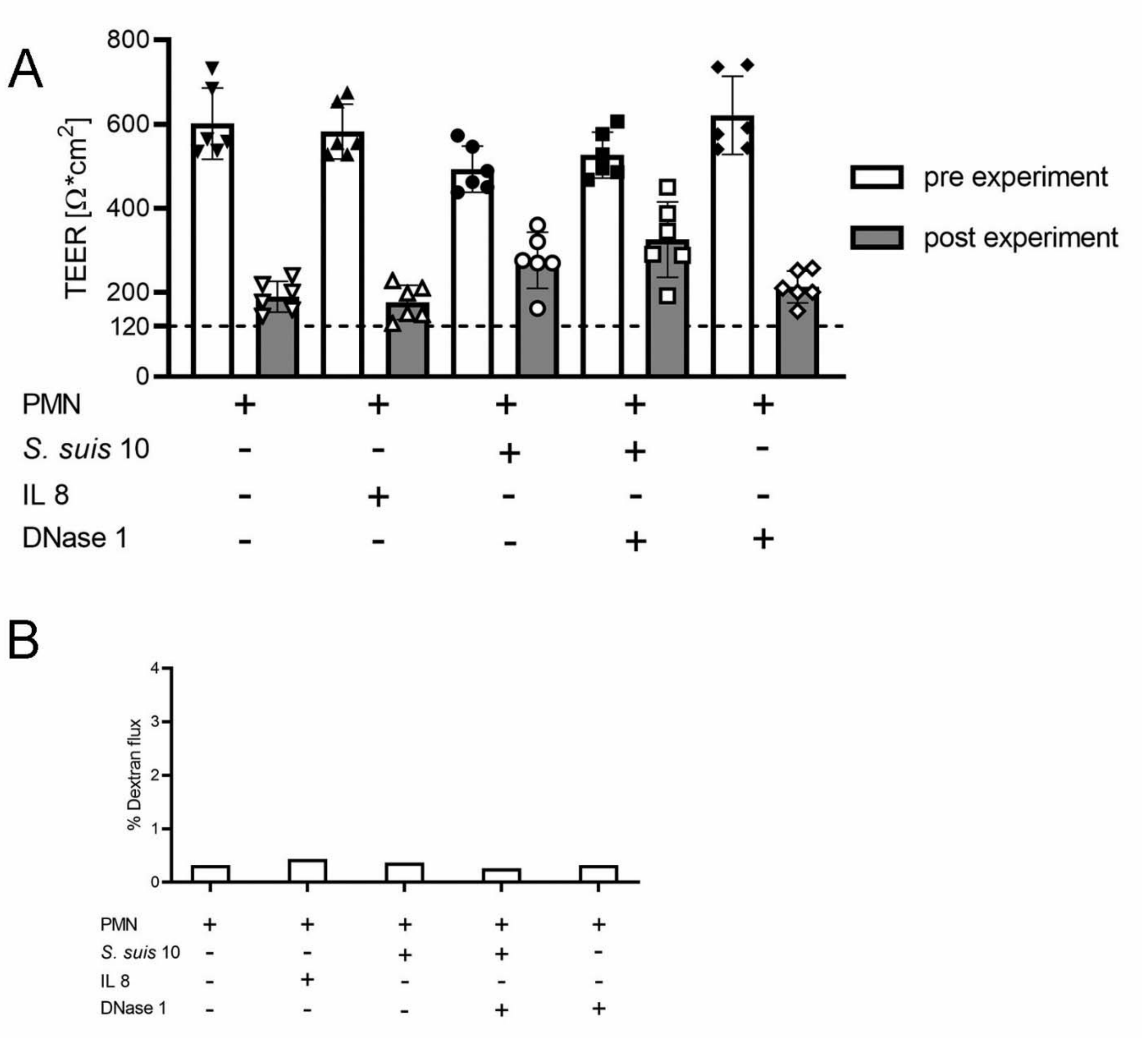

Fig. S10 The barrier integrity of PCP-R cells was tight during *S.suis*10 infection and neutrophil transmigration.

The presented data extend the data of figure 5 and belong to the experiment shown there. A) The transepithelial electrical resistance (TEER) was measured before the experiment and after 5h incubation. A limit of 120 Ω/cm^2 was set as lowest value to be used in this study. No value was below the limit after 5h of incubation. B) An additional test of the barrier integrity was performed by testing the dextran flux. In the upper compartment 0.05 mg of Dextran Texas Red (3000 MW; Invitrogen™, D3329) was added. After incubation of 4 hours the diffused amount of Dextran Texas Red in the lower compartment was determined. The fluorescence was measured in the Tecan

Plate Reader (Infinite 200 Pro) at a wavelength of 620nm (595nm excitation) and calculated compared to a twofold dilution standard series. Since dextran has an influence on neutrophils and phagocytosis and because the detected values confirmed values as shown already before [1,2], this experiment was performed only once.

References

1. Lauer, A. N. *et al.* Optimized cultivation of porcine choroid plexus epithelial cells, a blood–cerebrospinal fluid barrier model, for studying granulocyte transmigration. *Lab. Investig.* **99**, 1245–1255 (2019).

2. Schroten, M. *et al.* A novel porcine in vitro model of the blood-cerebrospinal fluid barrier with strong barrier function. *PLoS One* **7**, (2012).

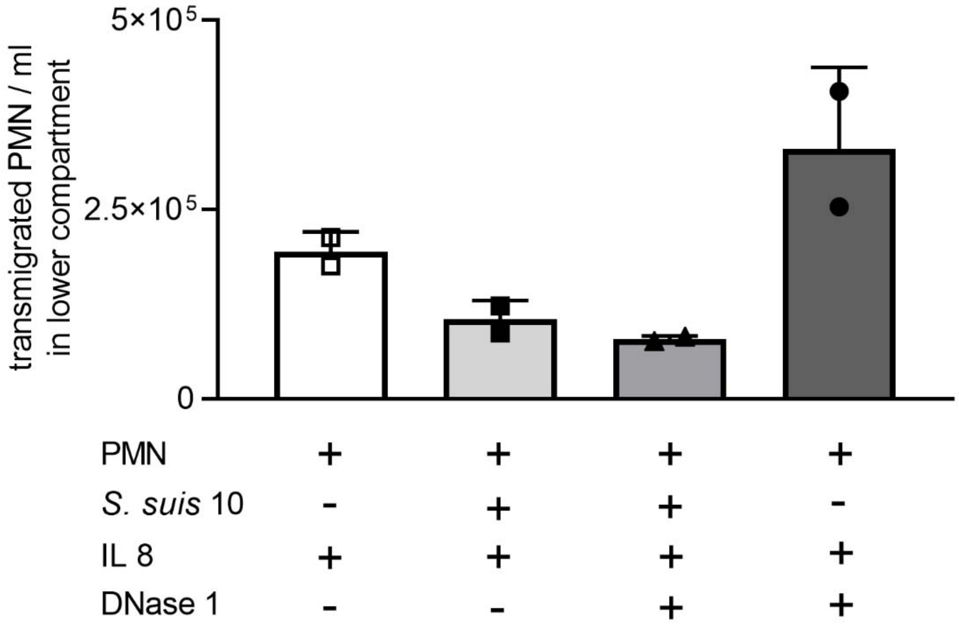

Fig. S11 Impact of IL8 on transmigration of isolated porcine neutrophils through a cell layer of porcine choroid plexus epithelial cells. To attract neutrophils 50ng interleukin 8 (IL8) (Recombinant Porcine IL-8/CXCL8, R&D Systems 535-IN-025) was added to all wells. 2 wells were enriched with 20 U DNase1 (Serva 18535.02) and 2 wells infected with 1-2 x10^2 CFU *S.suis*10 (non-infected wells, non-infected wells with DNase1, infected wells and infected wells with DNase1). Stimulation with 50 ng/ml interleukin 8 (IL 8) leads to an increase of neutrophil transmigration into the lower compartment in the cell culture system than without stimulation (Fig.5). *S.suis*10 or DNase1 were not increasing transmigration rate. N= 1 in duplicates.

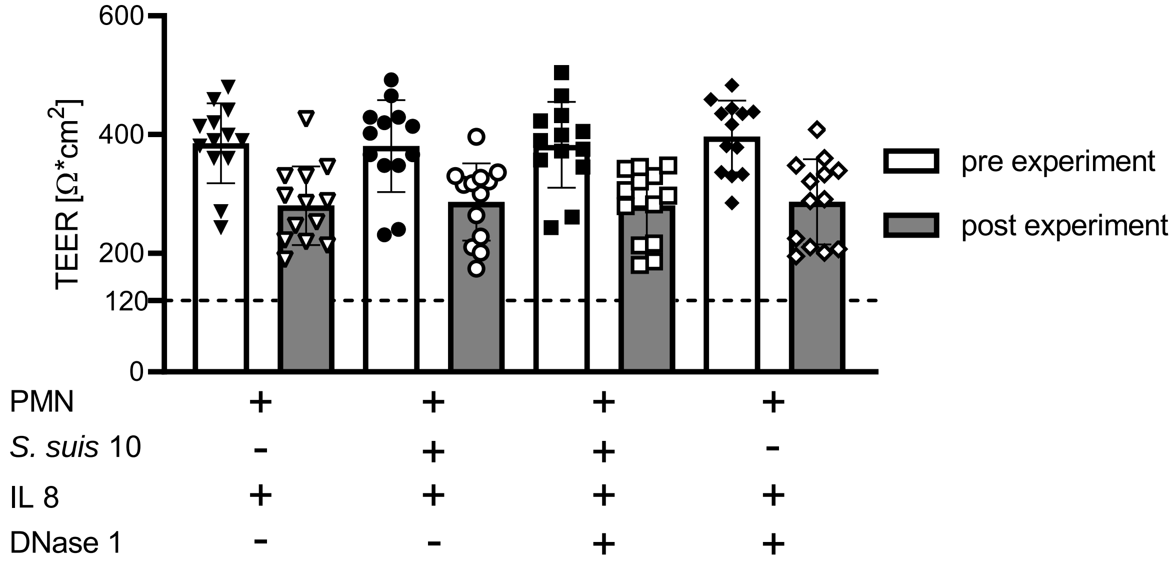

Fig. S12 The barrier integrity of PCP-R cells was tight during *S.suis*10 infection and neutrophil transmigration in the more physiological cell culture system.
The presented data extend the data of figure 7 and belong to the experiment shown there. The transepithelial electrical resistance (TEER) was measured before the experiment and after 4 h incubation. A limit of 120 Ω/cm^2 was set as lowest value to be used in this study. No value was below the limit after 4 h of incubation.

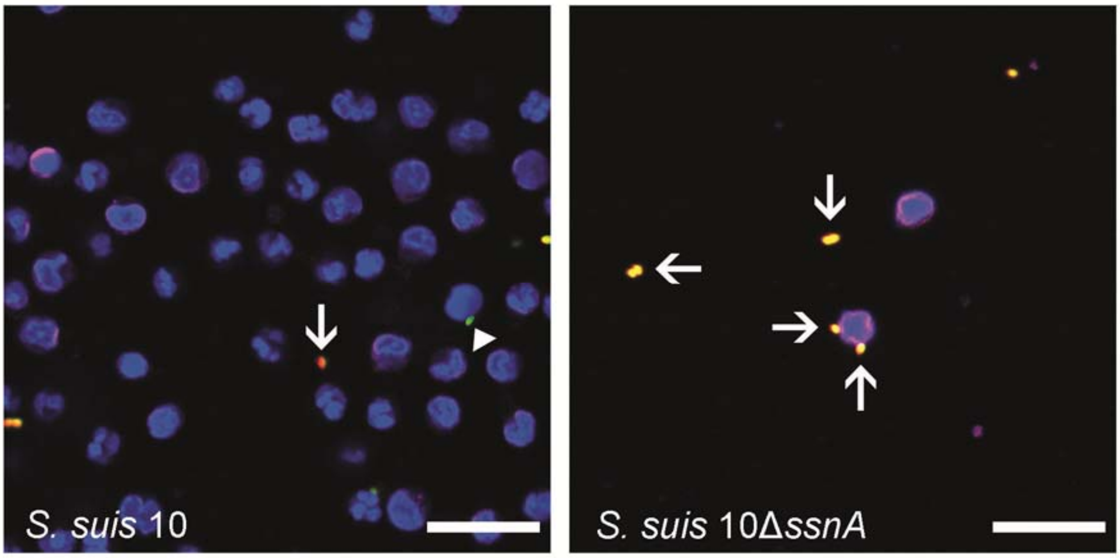

Fig. S13 More *S.suis* are found extracellularly than intracellularly in infected CSF of piglets with clinical meningitis.

(blue = DNA (Hoechst), magenta = DNA/ histone-1-complexes (NETs), red and green = extracellular *S.suis* (white arrows), green = intracellular *S.suis* (white arrow head). Representative pictures are shown (scale bar = 20 µm).
